# Pathogenicity of Serratia marcescens Strains in Honey Bees

**DOI:** 10.1128/mBio.01649-18

**Published:** 2018-10-09

**Authors:** Kasie Raymann, Kerri L. Coon, Zack Shaffer, Stephen Salisbury, Nancy A. Moran

**Affiliations:** a Department of Biology, University of North Carolina, Greensboro, North Carolina, USA; b Department of Integrative Biology, University of Texas at Austin, Austin, Texas, USA; University of Hawaii

**Keywords:** *Serratia marcescen*s, honey bee, opportunistic pathogen, virulence

## Abstract

Although few honey bee diseases are known to be caused by bacteria, pathogens of adult worker bees may be underrecognized due to social immunity mechanisms. Specifically, infected adult bees typically abandon the hive or are removed by guards. Serratia marcescens, an opportunistic pathogen of many plants and animals, is often present at low abundance in the guts of honey bee workers and has recently been isolated from Varroa mites and from the hemolymph of dead and dying honey bees. However, the severity and prevalence of S. marcescens pathogenicity in honey bees have not been fully investigated. Here we characterized three S. marcescens strains isolated from the guts of honey bees and one previously isolated from hemolymph. *In vivo* tests confirmed that S. marcescens is pathogenic in workers. All strains caused mortality when a few cells were injected into the hemocoel, and the gut-isolated strains caused mortality when administered orally. *In vitro* assays and comparative genomics identified possible mechanisms of virulence of gut-associated strains. Expression of antimicrobial peptide and phenoloxidase genes was not elevated following infection, suggesting that these S. marcescens strains derived from honey bees can evade the immune response in their hosts. Finally, surveys from four locations in the United States indicated the presence of S. marcescens in the guts of over 60% of the worker bees evaluated. Taken together, these results suggest that S. marcescens is a widespread opportunistic pathogen of adult honey bees and that it may be highly virulent under some conditions such as perturbation of the normal gut microbiota or the presence of Varroa mites that puncture the integument, thereby enabling entry of bacterial cells.

## Abstract (precis)

Recently, it has become apparent that multiple factors are responsible for honey bee decline, including climate change, pests and pathogens, pesticides, and loss of foraging habitat. Of the large number of pathogens known to infect honey bees, very few are bacteria. Because adult workers abandon hives when diseased, many of their pathogens may go unnoticed. Here we characterized the virulence of Serratia marcescens strains isolated from honey bee guts and hemolymph. Our results indicate that S. marcescens, an opportunistic pathogen of many plants and animals, including humans, is a virulent opportunistic pathogen of honey bees, which could contribute to bee decline. Aside from the implications for honey bee health, the discovery of pathogenic S. marcescens strains in honey bees presents an opportunity to better understand how opportunistic pathogens infect and invade hosts.

## INTRODUCTION

Despite extensive recent research on the reasons for global honey bee colony decline, no single factor has yet been implicated as a cause. In fact, most evidence suggests that honey bee colony decline stems from multiple factors, such as climate change, pests and pathogens, pesticides, and loss of foraging habitat (1, 2). Although honey bees have many known pathogens, few bacterial pathogens have been identified. The best-known bacterial diseases are European foulbrood and American foulbrood, which are caused by the Gram-positive species Melissococcus plutonius and Paenibacillus larvae and result from infection of larvae that die in the hive and leave diagnostic evidence of disease (3). Recognized pathogens of adult honey bees include Bacillus pulvifaciens (powdery scale disease) and two species of the genus *Spiroplasma* (May disease) (4). Adults with bacterial infections may be overlooked in many cases due to behaviors associated with social immunity, a phenomenon by which individual group members cooperate to combat disease transmission (5). For example, honey bees initiate precocious foraging when exposed to different stressors, including Varroa mites and *Nosema ceranae*, and specialized workers guard the nest entrance to attack or exclude infected nest mates (5). Therefore, it is possible that other bacterial pathogens of adult honey bees exist but have gone unnoticed because infected workers are largely excluded from the hive.

Opportunistic pathogens are organisms that can become pathogenic only in susceptible hosts, e.g., hosts with weakened immune systems or altered microbiome compositions (6). However, in most cases opportunistic pathogens coexist peacefully within their host and can live in several nonhost environments, making it difficult to identify them and determine the factors that lead to their pathogenicity. Many opportunistic pathogens are resistant to multiple antibiotics, and antibiotic treatment is often a precursor to infection (6). Honey bees experience many stressors that can disrupt their microbiota and/or alter their immune system, such as exposure to antibiotics (7, 8) and pesticides (9–11), increasing their risk of infection by opportunistic pathogens.

Serratia marcescens is a Gram-negative opportunistic pathogen of a wide range of animals, including humans and insects (12). In most animals, S. marcescens is virulent only when present in the bloodstream (12). In honey bees, S. marcescens has been detected in diseased larvae (13) and is sporadically found at low frequencies (typically <5% relative abundance) in the guts of adults, where it is considered a signifier of an atypical microbiome composition (7, 14–16). Recently, a S. marcescens strain (kz11) isolated from the honey bee gut was shown to cause increased mortality in adult bees following antibiotic or pesticide exposure (7, 17), and another strain, S. marcescens Ss1, was isolated from Varroa mites and the hemolymph of immobilized and dead bees, particularly in overwintered hives (18). Taking the data together, these studies suggest that S. marcescens is a common pathogen of honey bees. However, the virulence of honey bee-associated S. marcescens strains has not been experimentally tested.

Here we isolated and characterized three strains of S. marcescens from the guts of honey bees and compared them to Varroa/hemolymph-associated strain S. marcescens Ss1 (18). Using *in vivo* mortality assays, we showed that S. marcescens strains isolated from the gut and inoculated orally or injected into the hemolymph are highly virulent to honey bees. In contrast, we found that S. marcescens Ss1 exhibits virulence only when present in the hemolymph of honey bees, suggesting that it possesses mechanisms for virulence and infection that are different from those of the gut isolates. Using *in vitro* enzyme activity assays and comparative genomics, we identified potential virulence factors associated with the pathogenicity of gut-isolated strains. Furthermore, we investigated how S. marcescens infection impacts the honey bee immune response and found that honey bee-associated S. marcescens strains do not induce expression of antimicrobial peptides (AMPs) or phenoloxidase, suggesting that these strains possess mechanisms for evading the honey bee immune system. Overall, our results indicate that S. marcescens is an important and overlooked threat to honey bees.

## RESULTS

### S. marcescens is commonly found in the gut microbiome of honey bees.

**FIG 1:**
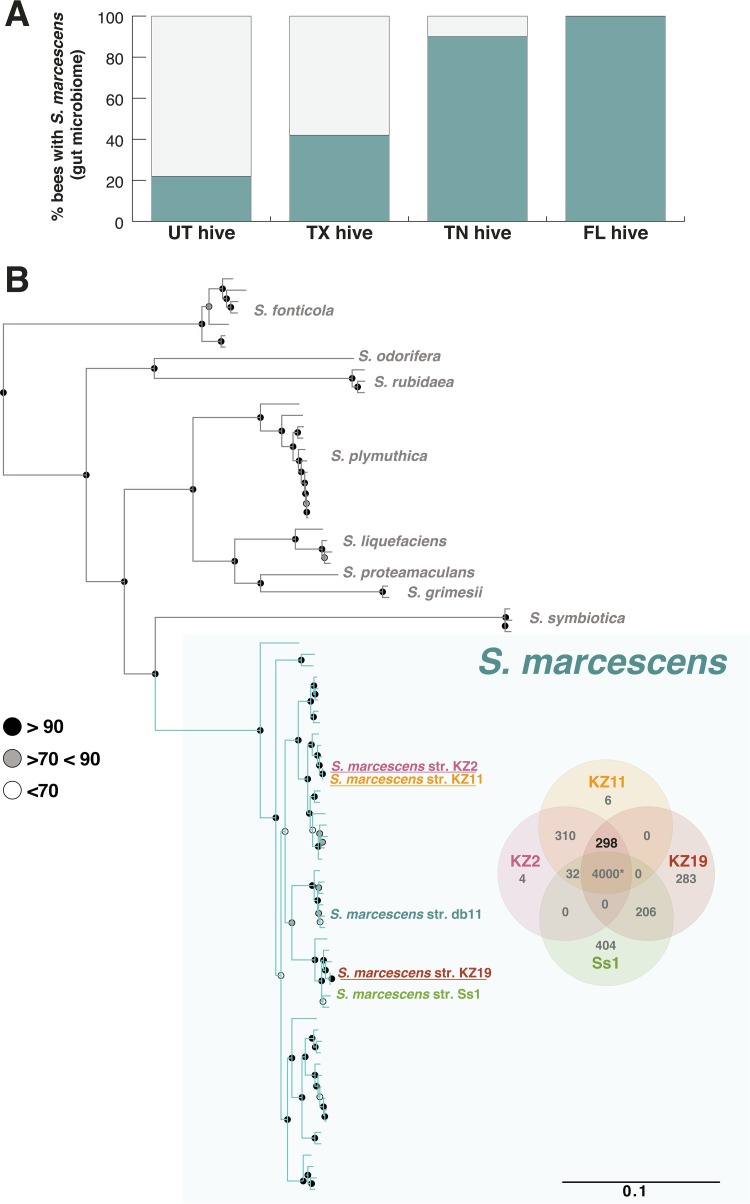
Frequency of S. marcescens in the gut microbiome of honey bees and the phylogeny of gut isolates. (A) Relative abundances of S. marcescens in the gut microbiome of honey bees from four locations: Utah (UT), Texas (TX), Tennessee (TN), and Florida (FL). (B) Phylogenetic tree of the genus *Serratia*. The three strains described here (kz2, kz11, and kz19), the other honey bee-associated strain (Ss1), and the *Drosophila*-associated strain (Db11) are shown in color. The tree was constructed using concatenated sequences of 125 proteins encoded by single-copy genes and analyzed with PhyML (GTR plus Gamm4) with 100 bootstrap replicates. Bootstrap values are represented by circles at each node (black, >90; gray, >70 and <90; white, <70). For the full tree, see Fig. S1. The Venn diagram on the right shows the number of genes shared by kz2, kz11, kz19, and Ss1.

We performed 16S rRNA profiling of the gut microbial community in honey bees from four locations: a hive kept on the University of Texas at Austin (UT) campus (*n* = 26); a commercial hive in Florida (*n* = 11); an organic hive in Tennessee (*n* = 21); and a long-established feral colony near Moab, Utah (*n* = 9). We identified S. marcescens in honey bees from all locations (Fig. 1). The proportions of bees containing S. marcescens in their gut ranged from 22% to 100% (Fig. 1A). However, the relative abundance was low (0.007% to 9.32%) in all S. marcescens-positive bees, suggesting that it can live as a bee gut commensal at low abundance (see Table S1 in the supplemental material). These results, coupled with previous gut microbiome studies of honey bees (7, 14–16), suggest that S. marcescens is frequently present at low abundance in the honey bee gut.

10.1128/mBio.01649-18.8 TABLE S1 Relative abundances of S. marcescens in sampled honey bee guts. Download Table S1, PDF file, 0.04 MB. Copyright © 2018 Raymann et al. 2018 Raymann et al. This content is distributed under the terms of the Creative Commons Attribution 4.0 International license.

10.1128/mBio.01649-18.1 FIG S1 Phylogenetic tree of *Serratia*. This tree represents the full version of the tree shown in Fig. 1, containing the GenBank assembly accession numbers for each strain. Download FIG S1, PDF file, 0.2 MB. Copyright © 2018 Raymann et al. 2018 Raymann et al. This content is distributed under the terms of the Creative Commons Attribution 4.0 International license.

The genomes of three S. marcescens strains (kz2, kz11, and kz19) isolated from the guts of honey bees were sequenced using Illumina MiSeq 2X300, assembled, and annotated (for more information about the genomes, see Table S2 and Materials and Methods). The kz11 strain was reported in a previous study (7) but was not sequenced or characterized. Phylogenetic and average nucleotide identity (ANI) analyses confirmed that these strains belong to the species S. marcescens (Table S2). Only one other honey bee-associated S. marcescens strain (Ss1) has been previously sequenced and characterized (18). One of our gut-isolated strains (kz19) is closely related to the Ss1 strain (99% ANI). The two other strains (kz2 and kz11) belong to a different clade, which consists of a mixture of human and environmental isolates, and these two strains are only 95% identical to Ss1 or ks19 based on ANI (Fig. 1B; see also Fig. S1 in the supplemental material). The genomes of kz11 and kz2 are 100% identical to one another based on ANI, differing only in gene content represented by a few accessory genes (Fig. 1B). The three kz strains share over 4,000 genes with Ss1 but also possess 298 genes that are not present in the Ss1 genome (Fig. 1B; see also Data Set S1 in the supplemental material). All of the honey bee-associated strains were 95% identical to the Db11 strain (19), a spontaneous streptomycin-resistant mutant derived from strain Db10 isolated from *Drosophila* (20); Db11 falls within a different subclade of S. marcescens (Fig. 1B; see also Table S2).

10.1128/mBio.01649-18.9 TABLE S2 Genome assembly and quality data for the S. marcescens strains sequenced in this study. Download Table S2, PDF file, 0.8 MB. Copyright © 2018 Raymann et al. 2018 Raymann et al. This content is distributed under the terms of the Creative Commons Attribution 4.0 International license.

10.1128/mBio.01649-18.10 DATA SET S1 List of the NCBI accession numbers and annotation for the 298 coding genes missing from the Ss1 genome. Gene clusters from Fig. 6 (see also Fig. S6 and S7) are highlighted in gray. Download Data Set S1, XLSX file, 0.1 MB. Copyright © 2018 Raymann et al. 2018 Raymann et al. This content is distributed under the terms of the Creative Commons Attribution 4.0 International license.

### S. marcescens is a pathogen of honey bees.

**FIG 2:**
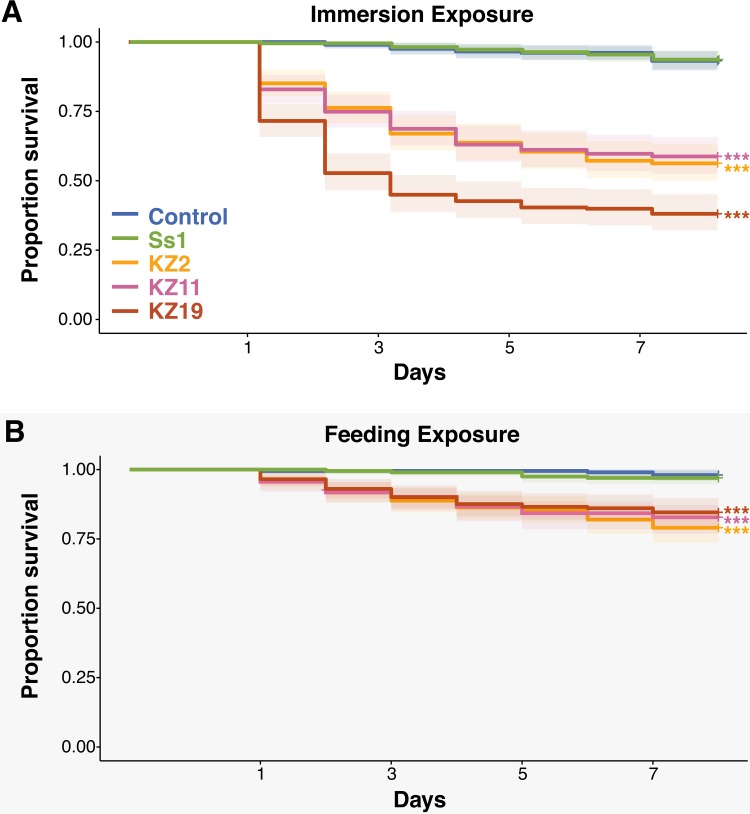
Survivorship of adult honey bee workers orally exposed to S. marcescens. Numbers of bees alive after oral exposure via the immersion method (A) and feeding method (B) are indicated. Survivorship was monitored and recorded each day for 7 days. The Kaplan-Meier survival curve was created using the “survival” package (21) in R (http://www.r-project.org/). Statistical analyses were performed using the coxph model implemented in that package. ***, *P* < 0.0001. See Fig. S2 for replicate experiments.

Using *in vivo* virulence assays, we found that all three S. marcescens bee gut isolates, Db11, and Ss1 are pathogenic to honey bees. The gut-isolated strains and Db11 showed high virulence when bees were exposed orally or through injection into the hemolymph. However, Ss1 was virulent only when present in the hemolymph. Adult worker bees were orally exposed to S. marcescens strains kz2, kz11, kz19, Db11, and Ss1. Two routes were used for oral exposure: (i) 500 µl of a bacterial solution applied to sterile pollen at an optical density (OD) of 1 (feeding exposure) and (ii) 10 µl of a bacterial sugar syrup solution applied to the body of each bee at an OD of 0.5 (immersion, which results in ingestion when bees clean one another). For both methods of exposure, kz2, kz11, kz19, and Db11 significantly decreased the survival rate of exposed bees compared to control bees, whereas Ss1 did not result in increased mortality (Fig. 2; see also Fig. S2). Exposure via immersion resulted in a much higher mortality rate (40% to 60%) (Fig. 2A; see also Fig. S2A and B) than exposure via feeding (10% to 20%) (Fig. 2B; see also Fig. S2C and D).

10.1128/mBio.01649-18.2 FIG S2 Survivorship of adult honey bee workers orally exposed to S. marcescens. Replicate experiments were performed using a second hive evaluating the number of bees alive after oral exposure via the immersion method (A and B) and the feeding method (C and D). Survivorship was monitored and recorded each day for 7 days. The Kaplan-Meier survival curve was created using the “survival” package (54) in R. Statistical analyses were performed using the coxph model implemented in the “survival” package (54) in R. **, *P* < 0.001; ***, *P* < 0.0001. Download FIG S2, PDF file, 0.8 MB. Copyright © 2018 Raymann et al. 2018 Raymann et al. This content is distributed under the terms of the Creative Commons Attribution 4.0 International license.

**FIG 3:**
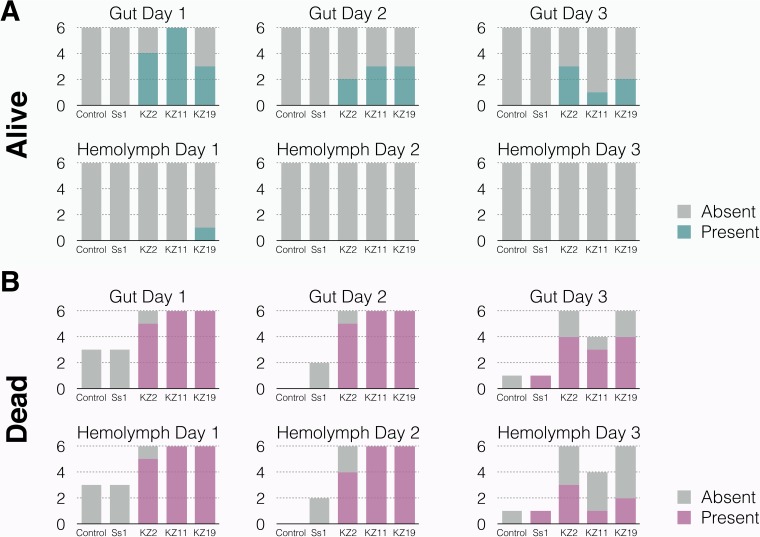
Detection of S. marcescens strains in the gut and hemolymph of living and dead bees. Numbers of living bees (A) and newly dead bees (B) possessing S. marcescens in their gut and hemolymph on days 1, 2, and 3 following oral exposure are indicated. Counts were based on CFU.

In other insects, it has been shown that S. marcescens is virulent only if it colonizes the hemolymph (12). To test if this is true in bees, adult workers were orally exposed to kz2, kz11, kz19, and Ss1 strains containing an E2 crimson fluorescent protein on an RSF1010 broad-host-range backbone with spectinomycin resistance (56). In order to maintain the plasmid, bees were fed a sterile sucrose solution containing 120 μl/ml of spectinomycin during the experiment. Plasmid-containing strains, which are blue/purple in color, were used to screen for the S. marcescens strains. For 3 days following exposure, six live bees were sampled from each group. In addition, up to six newly dead bees, if applicable (see Materials and Methods), were sampled. The gut and hemolymph of each bee were extracted and plated on LB agar containing 120 µg/ml spectinomycin. After 24 h of incubation at 30°C, the presence or absence of each S. marcescens strain was determined by the presence of blue/purple colonies on the plates. On day 1 following exposure, strains kz2, kz11, and kz19 were detected in at least 50% of the guts of living bees, but the numbers of bees sampled that possessed these strains in their gut decreased on days 2 and 3 (Fig. 3A). In contrast, Ss1 was never isolated from the guts of living bees (Fig. 3A). With the exception of a single kz19-exposed bee, none of the S. marcescens strains were observed in the hemolymph of living bees (Fig. 3A). In dead bees, kz2, kz11, and kz19 were isolated from both the gut and the hemolymph of almost all bees sampled on days 1 and 2. On day 3, kz2, kz11, and kz19 were detected in at least 50% of the guts of dead bees and in the hemolymph of some bees (Fig. 3B). Very few control and Ss1-exposed bees died during the experiment. Ss1 was not detected in the gut or hemolymph of living or dead bees on days 1 and 2, but Ss1 was detected in the gut and hemolymph of a single dead bee on day 3 (Fig. 3B). S. marcescens was never detected in the gut or hemolymph of control bees (Fig. 3).

**FIG 4:**
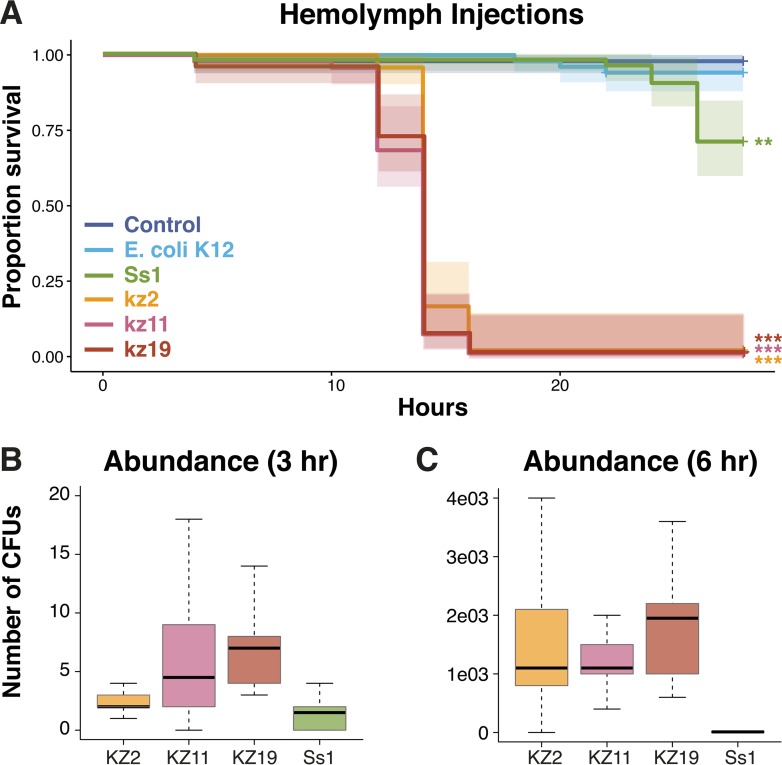
Survivorship of bees injected with S. marcescens. (A) Number of bees alive after hemolymph injection. Survivorship was monitored and recorded every 2 h for 24 h. The Kaplan-Meier survival curve was created using the “survival” package (21) in R. Statistical analyses were performed using the coxph model implemented in the “survival” package (21) in R. **, *P* < 0.001; ***, *P* < 0.0001. (B and C) S. marcescens CFU per microliter of hemolymph 3 h (B) and 6 h (C) after inoculation with ∼10 S. marcescens cells.

We performed hemolymph injection experiments to further investigate the virulence of the S. marcescens strains. Within 20 h, all bees injected with ∼10 cells of kz11, kz19, kz2, or Db11 died (Fig. 4A; see also Fig. S3). Injection with Ss1 also resulted in increased mortality, but the increase was less than that seen with the other S. marcescens strains. After 24 h, approximately 30% of Ss1-injected bees died (Fig. 4A). In a second experiment, injection with kz11, kz19, and kz2 again killed all bees within 20 h, but Ss1 exposure did not significantly increase mortality compared to controls (Fig. S3). In contrast, almost all bees that were injected with phosphate-buffered saline (PBS) only or with Escherichia coli strain K12 were still alive after 26 h (Fig. 4A; see also Fig. S3).

10.1128/mBio.01649-18.3 FIG S3 Survivorship of adult honey bee workers injected with S. marcescens. Replicate experiment using a second hive evaluating the number of bees alive after hemolymph injection. Survivorship was monitored and recorded every 2 h for 24 h. The Kaplan-Meier survival curve was created using the “survival” package (54) in R. Statistical analyses were performed using the coxph model implemented in the “survival” package (54) in R. **, *P* < 0.001; ***, *P* < 0.0001. Download FIG S3, PDF file, 0.1 MB. Copyright © 2018 Raymann et al. 2018 Raymann et al. This content is distributed under the terms of the Creative Commons Attribution 4.0 International license.

In order to determine how quickly the bee-associated strains replicate within the hemolymph, we evaluated the number of cells present in the hemolymph 3 and 6 h after the injections with ∼10 cells. At 3 h postinjection, the CFU count was similar to the number of cells injected (Fig. 4B; ∼10 cells per bee). At h 6, the average number of CFU of kz2, kz11, and kz19 was 1 × 10^3^ to 2 × 10^3^. In contrast, the number of Ss1 CFU did not significantly increase from h 3 to h 6 (Fig. 4C). The hemolymph of control bees produced no CFU.

In rich media, Ss1 grows more slowly than kz strains (Fig. S4). The slow growth of Ss1 compared to other S. marcescens strains was also reported in reference 18. Thus, Ss1 may also replicate more slowly within the hemolymph, resulting in a delay in virulence.

10.1128/mBio.01649-18.4 FIG S4 Growth curves of honey bee-associated S. marcescens strains. Strains were grown in LB media at 30°C for 48 h. Download FIG S4, PDF file, 0.5 MB. Copyright © 2018 Raymann et al. 2018 Raymann et al. This content is distributed under the terms of the Creative Commons Attribution 4.0 International license.

### Serratia marcescens infection does not cause an immune response in honey bees.

The honey bee innate immune response to S. marcescens infection was evaluated by assessing expression levels of the four honey bee antimicrobial peptide (AMP) gene families (abaecin, apidaecin, defensin, and hymenoptaecin) and the gene encoding the melanizing enzyme phenoloxidase following exposure to each strain. Bees were either injected with ∼10 cells or orally exposed using the immersion exposure method. The abdomen of the injected bees and the guts of the orally exposed bees were sampled 6 h after infection, and AMP and phenoloxidase gene expression were evaluated.

**FIG 5:**
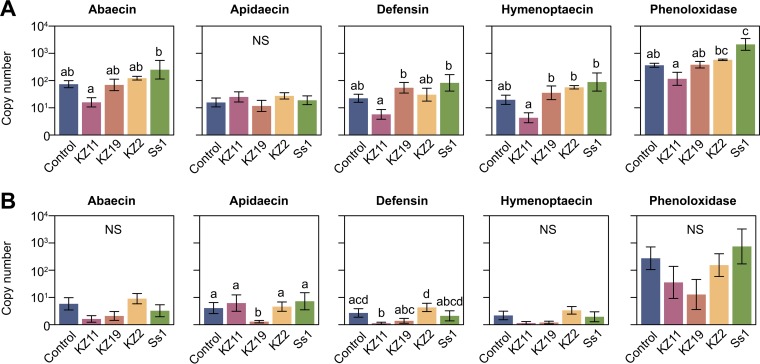
Immune-related gene expression in bees following S. marcescens inoculation. AMP and phenoloxidase transcript abundance in the abdomens of honey bee workers injected with S. marcescens (A) and the guts of honey bee workers exposed orally to S. marcescens 6 h postinfection (B). The bars in each graph show the copy number of each gene (± standard errors [SE]) per 0.5 μg of total RNA. For each treatment performed as described for panel A, different letters indicate significant differences in copy numbers as determined by a one-way analysis of variance (ANOVA) followed by *post hoc* comparisons performed using a Tukey-Kramer honestly significant difference (HSD) test (*P* < 0.05) in R. Significant differences between results of treatments performed as described for panel B were determined by a Kruskal-Wallis one-way ANOVA followed by a *post hoc* Dunn’s test to account for nonnormality of the data. NS, no significant differences were determined.

No significant changes in AMP expression were observed between bees injected with S. marcescens and control bees for any of the strains tested (Fig. 5A). However, bees injected with Ss1 showed increased expression of phenoloxidase compared to control bees or bees injected with kz11 or kz19 (Fig. 5A). Ss1-injected bees also showed higher expression of abaecin, defensin, and hymenoptaecin, but this difference was significant only in comparisons to bees injected with kz11 (Fig. 5A). Oral exposure of bees to different S. marcescens strains also generally failed to elicit significant changes in AMP or phenoloxidase expression compared to control bees (Fig. 5B). Notable exceptions to this were bees fed kz19 or kz11, which exhibited significant reductions in expression of apidaecin and defensin, respectively, compared to control bees (Fig. 5B).

### Virulence-associated enzymatic activity is detected in gut-isolated S. marcescens strains from honey bees.

Virulence is associated with several enzymatic activities, such as siderophore production, protease, chitinase, gelatinase, DNase, and hemolytic activity (22–27). Motility (e.g., swimming and swarming) has also been shown to play a role in virulence (28). We performed *in vitro* assays at three different temperatures (∼22°C [room temperature {RT}], 30°C, and 37°C) to determine if our strains and Ss1 possessed any of these activities. The three kz strains tested positive for all enzymatic and motility assays at 30 and 37°C after 24 and 48 h of incubation, with the exception of kz11, which exhibited hemolytic activity only after 72 h (Fig. S5). Protease activity, siderophore production, and gelatinase activity were observed for all kz strains at RT at 24 and 48 h (Fig. S5). In contrast, Ss1 exhibited DNase activity and swarm motility, the latter of which was observed only after incubation at 30 and 37°C (Fig. S5). DNase activity has previously been demonstrated in Ss1 (18).

10.1128/mBio.01649-18.5 FIG S5 *In vitro* assays of virulence-associated enzymatic activities. (A) Presence or absence of enzymatic activity for each S. marcescens strain at RT (∼22°C), 30°C, and 37°C after 24, 48, or 72 h of incubation. Filled circles represent the presence of a given activity, and empty circles indicate absence. (B) Photographs of the plates and culture tubes from each assay after 48 h of incubation at 30°C. Download FIG S5, PDF file, 12.8 MB. Copyright © 2018 Raymann et al. 2018 Raymann et al. This content is distributed under the terms of the Creative Commons Attribution 4.0 International license.

### S. marcescens gut isolates possess virulence genes that are missing from Ss1.

Because Ss1 did not exhibit virulence when ingested by bees under our experimental conditions, we investigated the genes shared by all kz strains but absent from Ss1 to identify genes potentially associated with the virulence of the gut isolates. Of the 298 genes unique to the kz strains (Data Set S1), 216 (72%) were found in clusters (sets of three or more consecutive genes that display conserved synteny). Because Db11 was also virulent to bees when ingested, we focused on the 13 clusters shared by all kz strains and the Db11 strain.

**FIG 6:**
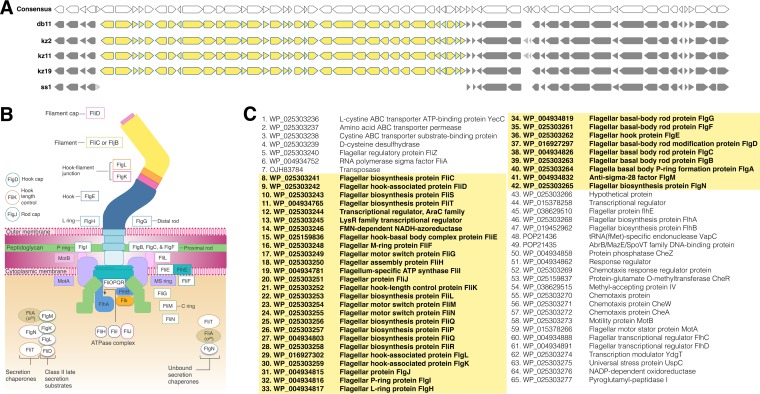
Flagellar components in honey bee-associated S. marcescens. (A) The flagellar gene cluster. Genes shown in yellow are missing from the Ss1 genome but are present in kz2, kz11, kz19, and Db11. Genes shared by all five strains are shown in dark gray. (B) Flagellar components of Salmonella enterica serovar Typhimurium. (Adapted from reference 29 with permission of the publisher.) Filled circles and boxes represent components present in Ss1. White circles and boxes represent components missing from the Ss1 genome. (C) List of accession numbers and annotations for genes shown in panel A.

One of the largest clusters missing from Ss1 encodes flagellar proteins (Fig. 6). Ss1 is missing 36 genes from the flagellar gene cluster which are present in all the kz strains as well as Db11 (Fig. 6), indicating that Ss1 does not have the ability to form fully functional flagella. In our motility assays, Ss1 did not exhibit swim motility but appeared to exhibit some swarming motility (Fig. S5). It is possible that Ss1 is not actually swarming but uses another mechanism that does not require flagella to move across surfaces, such as gliding or twitching (32). Two other clusters missing from Ss1 include genes involved in iron regulation and siderophore biosynthesis (Fig. S6), which is consistent with the finding that Ss1 does not produce siderophores (Fig. S5). The functional roles of the other 11 gene clusters missing from Ss1 are less clear, as they mostly include genes encoding hypothetical proteins, transcriptional regulators, and transporters (Fig. S7).

10.1128/mBio.01649-18.6 FIG S6 Gene clusters associated with iron acquisition missing from the Ss1 genome. Genes shared by all five strains are shown in dark gray. Genes missing from Ss1 but present in kz2, kz11, kz19, and Db11 are shown in purple. All other genes are colored depending on their presence in different strains. Download FIG S6, PDF file, 0.1 MB. Copyright © 2018 Raymann et al. 2018 Raymann et al. This content is distributed under the terms of the Creative Commons Attribution 4.0 International license.

10.1128/mBio.01649-18.7 FIG S7 Other gene clusters missing from the Ss1 genome. Genes shared by all five strains are shown in dark gray. Genes missing from Ss1 but present in kz2, kz11, kz19, and Db11 are shown in purple. All other genes are colored depending on their presence in different strains. Download FIG S7, PDF file, 0.5 MB. Copyright © 2018 Raymann et al. 2018 Raymann et al. This content is distributed under the terms of the Creative Commons Attribution 4.0 International license.

## DISCUSSION

Here we characterized the virulence of three S. marcescens strains isolated from the honey bee gut as well as S. marcescens Ss1 isolated from Varroa mites and the hemolymph of bees (18). We experimentally confirmed that Ss1 is virulent when present in the hemolymph, as suggested in a previous study (18). Moreover, we showed that the gut-isolated S. marcescens strains, when ingested and when injected into the body cavity, are highly virulent. We detected Ss1 in the gut of only one bee, which suggests that it is rarely capable of colonizing the gut, at least under our experimental conditions. We note that bees were exposed to very high doses of S. marcescens in our oral exposure experiments. In field-collected bees, S. marcescens is usually at low abundance, and virulence of gut-dwelling S. marcescens may depend on unusual conditions in which it becomes abundant in the gut.

The immersion method of oral exposure resulted in higher mortality than the feeding method. Bees immersed in the inoculum solution immediately clean themselves and each other and thus are rapidly exposed to large doses. In contrast, the amount ingested via feeding varies, depending on the rate and extent of consumption of the sugar syrup. Also, the immersion method potentially enables S. marcescens to invade through other routes, such as the respiratory tracheae or punctures in the integument. For our oral exposure and hemolymph injection experiments, we tested S. marcescens virulence on workers from hives that had not been treated with chemicals for >2 years. The mortality rate of bees infected with S. marcescens was previously shown to be much higher following exposure to the antibiotic tetracycline (7).

The immune response of honey bees and other insects consists of three levels of resistance: physical barriers, cell-mediated immunity, and cell-free humoral immunity (33). AMPs and phenoloxidase are key elements of humoral immunity (34). In general, we did not observe an increase in expression of the genes encoding the four bee AMPs or phenoloxidase following S. marcescens infection, suggesting that these strains possess mechanisms for evading (not triggering) the honey bee immune response, such as are known in some other animal pathogens (35). This general lack of immune induction potentially reflects the prior upregulation of AMP and phenoloxidase genes in response to colonization by the native gut microbial community (36), preventing us from observing further upregulation. Interestingly, we did observe a decrease in the expression of two AMP genes following oral exposure to two of the gut-isolated S. marcescens strains, consistent with an ability of some strains to suppress immune responses under certain conditions. We did not directly measure AMP or phenoloxidase activity in the present study. However, in a previous study of immune responses to gut microbiota in honey bees (36), transcript levels correlated with corresponding AMP abundance. Further investigation will be necessary to elucidate the molecular mechanisms underlying how different S. marcescens strains interact with the bee immune system as well as the impact of S. marcescens infection on expression of bee immune factors over longer durations of infection or under conditions in which the native microbiota are disrupted.

Successful colonization and pathogenicity may depend on the gut microbiome composition. Although we found that the gut-isolated S. marcescens strains were sometimes virulent when restricted to the gut (Fig. 3), virulence may require unusually high titers or proliferation near the gut wall, as might occur when the microbiome is disrupted. In the colonization experiments, bees were exposed to spectinomycin, prior to exposure to S. marcescens. Increased susceptibility to S. marcescens infection following tetracycline treatment (7) provides evidence supporting this hypothesis. Moreover, some bee gut community members possess antibacterial weaponry, potentially contributing to bee defense against bacterial pathogens (37). For example, one of the core bee gut bacterial species, Snodgrassella alvi, possesses type VI secretion systems that deliver antibacterial toxins and that kill members of other bacterial species (37).

As in other animals (12), S. marcescens virulence in honey bees might correlate with the ability of the strains to colonize the gut and subsequently penetrate the gut wall and enter the body cavity, causing septicemia. We identified S. marcescens in the hemolymph of most dead bees orally inoculated with the gut-isolated strains, whereas it was rare in the hemolymph of live bees (Fig. 3). While this suggests that pathogenicity involves invasion of the body cavity via the gut, another possibility is postmortem proliferation in hemolymph. In contrast, Ss1, which was isolated from hemolymph of diseased honey bees (18), appears unable to colonize the gut, suggesting that its mode of infection is different from that of the gut-isolated strains.

Under conditions of injection into the hemolymph, 100% of bees injected with the gut-isolated S. marcescens strains or Db11 died within 20 h, and 30% of bees injected with Ss1 died within 26 h. The delay in mortality of bees injected with Ss1 may reflect its low replication rate in the hemocoel (Fig. 4). Despite this slower replication, our results confirm that Ss1 is lethal to honey bees when present in the hemocoel. The fact that Ss1 colonizes only the hemocoel supports the hypothesis that Ss1 is transmitted by mites (18). We did not test whether the gut isolates are transmitted by Varroa mites. However, wounds from mite bites could enable these strains to access the hemolymph, which would result in death within 1 day.

Comparing gene sets possessed by S. marcescens kz2, kz11, kz19, Db11, and Ss1 strains allowed us to identify candidate genes associated with the virulence of the gut isolates. Although kz19 is more closely related to Ss1, it shares 298 genes uniquely with kz2 and kz11 (Fig. 1), which had similar pathogenicity attributes in our experiments. Among these genes, the flagellar genes and genes involved in iron acquisition are promising virulence candidates, and both have been shown to be important for pathogenicity in other bacteria (28, 38–40). The loss of flagellar genes from Ss1 is particularly interesting, because this loss is frequently observed in obligate endosymbionts and intracellular pathogens (41). Flagella are costly to maintain as their production significantly decreases bacterial growth (42). Transmission of Ss1 by Varroa mites (18) might make motility unimportant for transmission, resulting in the loss of flagellar genes.

S. marcescens strains are widespread in honey bee guts, and our results show that oral exposure to these strains can lead to lethal infections. Disruption of the gut microbiome appears to facilitate invasion by S. marcescens (7). Because honey bees are frequently exposed to factors that could disrupt the gut microbiome, S. marcescens infection may be common and potentially contributes to colony losses. Entry of even a few cells into the hemocoel can be lethal, supporting the possibility that S. marcescens pathogenicity is exacerbated by Varroa mites (18). In addition, the discovery of S. marcescens strains that can be pathogenic in honey bees, which represent a tractable model system, presents an opportunity to study how and when opportunistic pathogens are able to invade and kill their hosts.

## MATERIALS AND METHODS

### Survey of S. marcescens in the honey bee gut microbiome.

Honey bees from a hive located on the UT campus, a feral hive from Utah, a commercial hive from Florida, and an organic hive from Tennessee were dissected, and DNA was extracted using the phenol chloroform bead-beading protocol described in reference 43. Community profiling was performed using the V4 region of the 16S rRNA gene with primers 515F and 806R as described previously (44). Reaction products were purified with AMPure XP beads (Beckman Coulter). The resulting amplicons were subjected to Illumina sequencing on the MiSeq platform (2 × 250 sequencing runs) at the Genome Sequencing and Analysis Facility (GSAF) at UT. Reads were processed in QIIME (45). FASTQ files were filtered for quality by allowing a minimum Phred quality score of Q20. Forward and reverse Illumina reads were joined, and chimeric sequences were removed using the usearch6.1 detection method. Operational taxonomic units (OTUs) were clustered at 97% with the UCLUST algorithm using the July 2017 release of the SILVA (46) reference database. Sequences that did not match the SILVA data set were subsequently clustered into *de novo* OTUs with UCLUST. Unassigned, mitochondrial, and chloroplast reads were removed from the data set. All OTUs present in less than 0.1% abundance were removed. The samples were rarefied at a depth of 15,000 reads per sample. For each bee, presence or absence of S. marcescens was evaluated based on the taxonomic assignment of the OTUs; e.g., if a bee had an OTU assigned to the species S. marcescens after filtering, it was marked as positive. The relative abundance of S. marcescens per bee was determined based on the percentage of S. marcescens reads among the rarefied 15,000 total reads per sample (see Table S1 in the supplemental material).

### Isolation of Serratia marcescens from honey bees.

Honey bee guts were surveyed for the presence of S. marcescens. Using workers from a package of bees from a treatment-free apiary, we extracted the guts of hundreds of bees (of these, 21 were sampled for 16S rRNA gene sequencing; see Tennessee [TN] data in Fig. 1). Gut extractions were performed as described in reference 7. The extracted guts were homogenized individually using sterile pestles and kept in 20% glycerol at −80°C. The stocks were plated onto LB agar plates and incubated at 30°C for 24 h (resident gut bacteria do not grow on this medium). Bacterial colonies that grew within 24 h were picked and replated. The isolated bacteria were PCR amplified using universal 16S rRNA gene primers 27F (5′-AGAGTTTGATCCTGGCTCAG-3′) and 1507R (5′-TACCTTGTTACGACTTCACCCCAG-3′). The PCR products were submitted for Sanger sequencing at the GSAF at UT. Three strains were identified with >97% identity to S. marcescens based on a BLASTn search against the NCBI nonredundant database (https://www.ncbi.nlm.nih.gov/).

### DNA extraction and genome sequencing.

DNA was extracted using the phenol chloroform bead-beading protocol described in reference 43, with the following modifications: cetyltrimethylammonium bromide (CTAB) and 2-mercaptoethanol were not used, the samples were bead beaten for only 2 min total, and the lysate was incubated at 56°C for 1 h. The resulting DNA was then submitted for Illumina MiSeq paired-end 2 × 300 sequencing at the GSAF at UT, resulting in 1,281,027 (strain kz2), 1,994,790 (strain kz11), and 915,651 (strain kz19) reads. Reads were assembled using CLC Workbench (see Table S2 for assembly details) with default parameters, producing a total of 20 (kz2), 21 (kz11), and 24 (kz19) contigs. Genomes were annotated with the NCBI Prokaryotic Genome Annotation Pipeline with GeneMarkS+ version 4.3 (https://www.ncbi.nlm.nih.gov/genome/annotation_prok/).

### Phylogenetic analysis.

A total of 102 *Serratia* genomes were downloaded from NCBI as well as a genome of Yersinia enterocolitica, which was used as an outgroup. Coding sequences were translated into protein sequences using an in-house script. Usearch (47) was used to identify orthologs (based on best reciprocal hits) with thresholds of 70% sequence identity and 80% length conservation. In-house scripts were used to identify single-copy protein families present in 100% of the genomes. A total of 125 single-copy gene families were identified. These protein sequences were aligned using MAFFT v7 (48) and concatenated. The concatenated alignment was used to build a maximum likelihood phylogeny using PhyML (49) with the following parameters: GTR, Gamma4, and 100 bootstrap replicates.

### Survival assays.

Workers of random ages were taken from a single hive on the UT campus. Bees were immobilized at 4°C and split into the following seven treatment groups, each containing about 200 workers: (i) control, (ii) E. coli K12 and S. marcescens, (iii) strain kz2, (iv) strain kz11, (v) strain kz19, (vi) strain Ss1, and (vii) strain Db11.

#### Feeding exposure.

Each group was divided into 5 cup cages containing approximately 40 bees. A solution of bacterial cells in PBS (or PBS alone for controls) at an OD of 1 was applied to irradiated bee bread and placed in each cup cage. Bees were monitored for survival every day for 1 week. The experiment was then replicated using a second hive on the UT campus.

#### Immersion exposure.

Groups of bees were immobilized at 4°C in a 50-ml Falcon tube, and 10 µl of a 1:1 solution of bacterial cells at an OD of 0.5 in PBS and sterile sucrose (or PBS and sucrose alone for controls) was added per bee. The tube was lightly shaken for 15 s before the bees were placed into cup cages. Each group was divided into 5 cup cages containing approximately 40 bees. Bees were monitored for survival every day for 1 week. The experiment was then replicated using a second hive on the UT campus.

#### Hemolymph injections.

A PBS bacterial solution (1 ml) at an OD of 1 was serial diluted to 10^−4^, and 1 µl of this solution (approximately 10 bacterial cells) was injected into the abdomen of worker bees using a fine-tipped glass capillary needle. Control bees were injected with 1 µl of sterile PBS. Each group was divided into 4 cup cages with approximately 12 bees per cup. Bees were monitored for survival for 24 h, and the number of dead bees for each treatment group was recorded approximately every 2 h. During the experiments, bees were kept in a 35°C incubator with 95% humidity to mimic hive conditions. The experiment was then replicated using a second hive on the UT campus.

### Hemolymph colonization.

Workers of random ages were taken from a single hive on the UT campus. Bees were immobilized at 4°C and split into five groups consisting of a control and S. marcescens strains kz2, kz11, kz19, and Ss1. During the experiments, bees were kept in a 35°C incubator with 95% humidity to mimic hive conditions. For each bacterial treatment, a PBS bacterial solution at an OD of 1 (1 ml) was serially diluted to 10^−4^, and 1 µl of this solution (approximately 10 bacterial cells) was injected into the abdomen of 10 worker bees per group using a fine-tipped glass capillary needle. Control bees were injected with 1 µl of sterile PBS. At 3 and 6 h postinjection, hemolymph was collected from the thorax of each bee using a fine-tipped glass capillary needle. Aliquots (0.5 µl) of undiluted and 1:100-diluted hemolymph were plated onto LB agar. After overnight incubation at 30°C, the number of CFU per agar plate was determined to calculate the number of S. marcescens cells per microliter of hemolymph.

### Gut colonization and septicemia tests.

An E2-Crimson fluorescent protein on an RSF1010 broad-host-range backbone with spectinomycin resistance (56) was transformed into S. marcescens strains kz2, kz11, kz19, and Ss1. In brief, an E. coli donor strain bearing pBTK570 was mixed in a 1:1 OD ratio with the recipient S. marcescens strain and incubated overnight on LB plates supplemented with diaminopimelic acid (DAP). Transconjugant S. marcescens strains were selected with 180 μg/ml spectinomycin, and visible E2-Crimson expression confirmed successful transconjugants.

Workers of random ages were taken from a single hive on the UT campus. Bees were immobilized at 4°C and split into the following five groups: (i) control and S. marcescens, (ii) strain kz2, (iii) strain kz11, (iv) strain kz19, and (v) strain Ss1. Each group was divided into 3 cup cages with approximately 20 bees per cup. Bees were fed a sterile sucrose solution containing 120 µg/ml spectinomycin for 2 days prior to S. marcescens exposure as well as for all 3 days of the experiment. During the experiments, bees were kept in a 35°C incubator with 95% humidity to mimic hive conditions. Bees were exposed to the S. marcescens strains using the immersion method described above. Each day, six live bees were sampled from each group as well as six dead bees if applicable. Dead bees were sampled <2 h postmortem. In the case of control and Ss1-exposed bees, only five and six bees, respectively, died during the 3-day experiment. The gut and hemolymph of each bee were extracted. In brief, guts for individual bees were homogenized in 1-ml sterile PBS and plated on LB agar containing 120 µg/ml spectinomycin using an inoculation loop. The hemolymph was collected from each bee, and 0.5-µl aliquots of undiluted hemolymph were plated on LB agar containing 120 µg/ml spectinomycin. Plates were incubated at 30°C for 36 h and then screened for the presence of blue/purple colonies.

### Bacterial growth curves.

S. marcescens cells were cultured in 24-well plates containing LB medium. The plates were incubated in a plate reader (Tecan) at 30°C for 48 h. Optical density was measured at 600 nm every 0.5 h, and plates were shaken for 2 min before each measurement.

### Immune response to Serratia marcescens infection.

Worker bees of random ages were taken from a single hive on the UT campus. Bees were immobilized at 4°C and split into the following five groups: (i) control and S. marcescens, (ii) strain kz2, (iii) strain kz11, (iv) strain kz19, and (v) strain Ss1. Half of the bees were fed (immersion exposure), and the other half were injected with the S. marcescens strains as described above under “Survival assays.” During the experiments, bees were kept in a 35°C incubator with 95% humidity to mimic hive conditions. At 6 h after S. marcescens exposure, the abdomens of the injected bees and the guts of the fed bees were dissected (10 bees per group) and transferred to an RNase-free 1.5-ml tube. Total RNA was then extracted from each sample using 1 ml of TRIzol (Life Technologies) according to the manufacturer’s instructions, with the addition of a 1-min bead-beating step using ∼0.5 ml of 0.1-mm-diameter silica zirconia beads (BioSpec Products). Samples were resuspended in 50 μl of nuclease-free water and were then treated with Promega RQ1 RNase-Free DNase according to the manufacturer’s protocol. RNA samples were quantified using a Qubit 2.0 fluorometer and a Quant-iT RNA BR kit. cDNA was synthesized according to the qScript cDNA synthesis kit protocol (Quanta Biosciences) using 0.5 μg of RNA per sample as the template. cDNA samples were diluted 1:10 with nuclease-free water for use as the template for quantitative PCR (qPCR).

Expression levels in unexposed and exposed bees were determined for the genes encoding abaecin (using primers from reference 50), apidaecin (51), defensin (50), hymenoptaecin (50), and phenoloxidase (50). In brief, PCR products were amplified from cDNA, purified, and quantified to determine copy number and then serially diluted to produce standard curves for each gene using qPCR. qPCR reactions were carried out with 10-μl reaction mixtures on an Eppendorf Mastercycler ep realplex system using iTaq Universal SYBR green Supermix (Bio-Rad) according to the manufacturer’s instructions. Expression levels were measured in triplicate for each biological replicate and normalized against the RPS5 housekeeping gene (52).

### *In vitro* assays of virulence factors.

Each S. marcescens strain was grown in liquid LB overnight. Overnight (∼36-h) cultures were then washed three times with PBS and resuspended in PBS to an OD of 1/ml.

#### Protease activity assay.

Milk agar plates were made as follows: 2.5 g yeast extract and 7.5 g agar were dissolved in 350 ml of water; 15 g of milk powder was dissolved in 150 ml of water; both solutions were then autoclaved, cooled, mixed, and poured into plates. A 1-µl volume of a bacterial PBS solution at an OD of 1 was spot plated (four spots) onto a milk agar plate and incubated at RT (∼22°C), 30°C and 37°C. Protease activity was assessed at 24 and 48 h.

#### Hemolytic activity assay.

Blood agar plates were made using heart infusion agar (HIA) and 5% sheep blood. S. marcescens strains (bacteria at an OD of 1 in PBS) were streaked unto blood agar plates and incubated at RT, 30°C, and 37°C. Hemolytic activity was assessed after 48 and 72 h.

#### Swimming and swarming motility assays.

Swim plates were made as follows. Peptone (5 g), yeast extract (3 g), and agar (0.3%) (3 g) were dissolved in 1 liter of water, autoclaved, and poured into plates; plates were then allowed to solidify and used the same day. Bacterial PBS solutions at an OD of 1 were diluted to 10^3^, and 1 µl of this dilution was stab inoculated into the center of the swim plate. Swarm plates were made using the same ingredients as the swim plates but with 5 g of agar (0.5%). The plates were allowed to dry overnight. Bacterial PBS solutions at an OD of 1 were diluted as described above, and 1 µl was spotted in the center of the plate using a sterile flat toothpick. Swim and swarm plates were incubated at RT, 30°C, and 37°C and assessed at 24 and 48 h.

#### Gelatinase hydrolysis test.

Gelatinase culture tubes were prepared as follows: 5 g of peptone, 3 g of yeast extract, and 120 g of gelatin were dissolved in 1 liter of water and autoclaved; the autoclaved medium was then dispensed into culture tubes and cooled overnight at 4°C. The gelatin was stabbed (toothpick) with bacteria (bacteria at an OD of 1 in PBS) and incubated at RT, 30°C, and 37°C. At 24 and 48 h after incubation, the gelatin was cooled and the assay was read.

#### DNase activity assay.

Plates were made using BD Difco DNase test agar with methyl green. S. marcescens strains (bacteria at an OD of 1 in PBS) were streaked unto DNase agar plates and incubated at RT, 30°C, and 37°C. DNase activity was assessed at 24 and 48 h.

#### Siderophore detection.

Siderophore detection plates were made as follows. Chromeazurol S (CAS) (60.5 mg) was dissolved in 50 ml water, and 72.9 mg CTAB was dissolved in 40 ml water. Those two solutions were then mixed with 10 ml of a 1 mM FeCl_3_ hexahydrate and 10 mM HCl solution and added to LB agar medium (the recipe for 1 liter was used, and the medium was dissolved in 900 µl of water). The pH was adjusted to 6.8 and then autoclaved. S. marcescens strains (bacteria at an OD of 1 in PBS) were streaked unto the siderophore detection plates and incubated at RT, 30°C, and 37°C. Siderophore production was assessed at 24 and 48 h.

#### Chitinase activity assay.

Chitin powder (40 g) was dissolved in 500 ml of concentrated hydrochloric acid and continuously stirred at 4°C for 1 h. The hydrolyzed chitin was washed several times with distilled water to remove the acid and to bring the pH to the range of 6 to 7. The colloidal chitin was then filtered and stored at 4°C. Chitin plates were made as follows: 3 g of MgSO_4_ heptahydrate, 3 g of (NH_4_)_2_SO_4_, 2 g of KH_2_PO_4_, 1 g of citric acid, 15 g of agar, 4.5 g of coilloidal chitin, and 15 g of bromocresol purple were dissolved in 1 liter of water; 200 µl of Tween 20 was then added; the pH was adjusted to 4.7; and the medium was autoclaved. S. marcescens strains (bacteria at an OD of 1 in PBS) were streaked unto the chitin plates and incubated at RT, 30°C, and 37°C. Chitinase activity was assessed at 24 and 48 h.

### Comparative genomics.

Protein families were clustered using SiLix (53) with 85% identity and 70% length conservation thresholds. The identified protein families were then aligned, and HMMER (54) profiles were constructed. The HMMER profiles were used to search a local database of the S. marcescens kz2, kz11, kz19, and Ss1 genomes in order to confirm their presence or absence in each genome. Among the 328 unique genes identified (see Data Set S1 in the supplemental material), missing gene clusters were further analyzed. Gene clusters were defined as three or more consecutive genes that displayed conserved genome synteny. All gene clusters unique to kz2, kz11, and kz19 were analyzed and visually inspected using Geneious version 11.0.4 (55). Gene clusters were subjected to local BLAST search against S. marcescens Db11 using Geneious version 11.0.4 (55).

### Data availability.

The genomes of S. marcescens strains kz2, kz11, and kz19 are available on NCBI (accession numbers PQGJ00000000, PQGI00000000, and PQGK00000000). 16S rRNA gene reads are deposited with NCBI Sequence Read BioProject under project number PRJNA483763.

## Note Added after Publication

In the originally published version, reference 56 was omitted. The reference was added to the References list and is now cited on pages 4 and 12.
