# Correction for Raymann et al., “Pathogenicity of Serratia marcescens Strains in Honey Bees”

**DOI:** 10.1128/mBio.02855-18

**Published:** 2019-02-05

**Authors:** Kasie Raymann, Kerri L. Coon, Zack Shaffer, Stephen Salisbury, Nancy A. Moran

**Affiliations:** a Department of Biology, University of North Carolina, Greensboro, North Carolina, USA; b Department of Integrative Biology, University of Texas at Austin, Austin, Texas, USA

## AUTHOR CORRECTION

Volume 9, no. 5, e01649-18, 2018, https://doi.org/10.1128/mBio.01649-18. This article was published on 9 October 2018 with reference 56 omitted. The reference and accompanying citations were updated in the current version on 2 January 2019.

