# Supplementary material for: Pathogenicity of Serratia marcescens Strains in Honey Bees
Source: mBio. 2018 Oct 9;9(5):e01649-18. doi: 10.1128/mBio.01649-18 (PMC6178626; doi:10.1128/mBio.01649-18)
Supplement: TABLE S1 [file mbo005184101st1.pdf]

Table S1. Relative abundance of *S. marcescens* in sampled honey bee guts.

Sample	Percent relative abundance of *Serratia marcescens*
Tennessee bee 1	0.00
Tennessee bee 2	0.01
Tennessee bee 3	0.04
Tennessee bee 4	0.07
Tennessee bee 5	0.23
Tennessee bee 6	0.01
Tennessee bee 7	0.00
Tennessee bee 8	0.70
Tennessee bee 9	0.01
Tennessee bee 10	0.16
Tennessee bee 11	0.09
Tennessee bee 12	0.80
Tennessee bee 13	3.19
Tennessee bee 14	0.12
Tennessee bee 15	0.57
Tennessee bee 16	1.59
Tennessee bee 17	1.85
Tennessee bee 18	1.47
Tennessee bee 19	3.29
Tennessee bee 20	3.41
Tennessee bee 21	3.93
Florida bee 1	2.46
Florida bee 2	0.03
Florida bee 3	0.75
Florida bee 4	0.30
Florida bee 5	0.39
Florida bee 6	0.06
Florida bee 7	0.01
Florida bee 8	0.01
Florida bee 9	0.33
Florida bee 10	0.01
Florida bee 11	0.47
Utah bee 1	0.00
Utah bee 2	0.00
Utah bee 3	0.00
Utah bee 4	0.00
Utah bee 5	0.03
Utah bee 6	0.01
Utah bee 7	0.00
Utah bee 8	0.00
Utah bee 9	0.00
Texas bee 1	9.32
Texas bee 2	1.80
Texas bee 3	0.62
Texas bee 4	0.42
Texas bee 5	0.24
Texas bee 6	0.18
Texas bee 7	0.10
Texas bee 8	0.08
Texas bee 9	0.04
Texas bee 10	0.04
Texas bee 11	0.02
Texas bee 12	0.02
Texas bee 13	0.00
Texas bee 14	0.00
Texas bee 15	0.00
Texas bee 16	0.00
Texas bee 17	0.00
Texas bee 18	0.00
Texas bee 19	0.00
Texas bee 20	0.00
Texas bee 21	0.00
Texas bee 22	0.00
Texas bee 23	0.00
Texas bee 24	0.00
Texas bee 25	0.00
Texas bee 26	0.00
