# Supplementary figures and images for: Pathogenicity of Serratia marcescens Strains in Honey Bees
Source: mBio. 2018 Oct 9;9(5):e01649-18. doi: 10.1128/mBio.01649-18 (PMC6178626; doi:10.1128/mBio.01649-18)

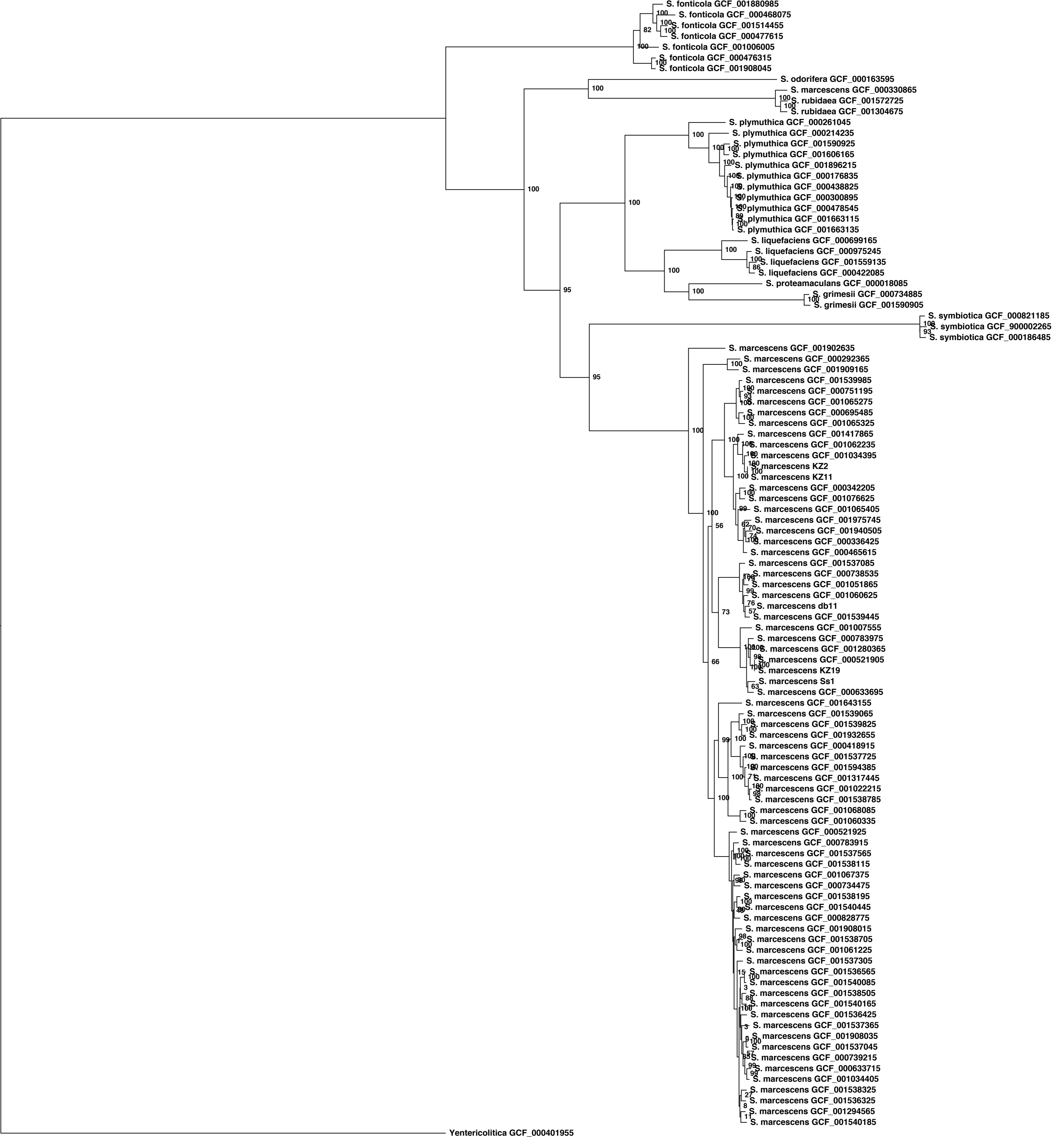

Supplement: FIG S1 [file mbo005184101sf1.pdf]

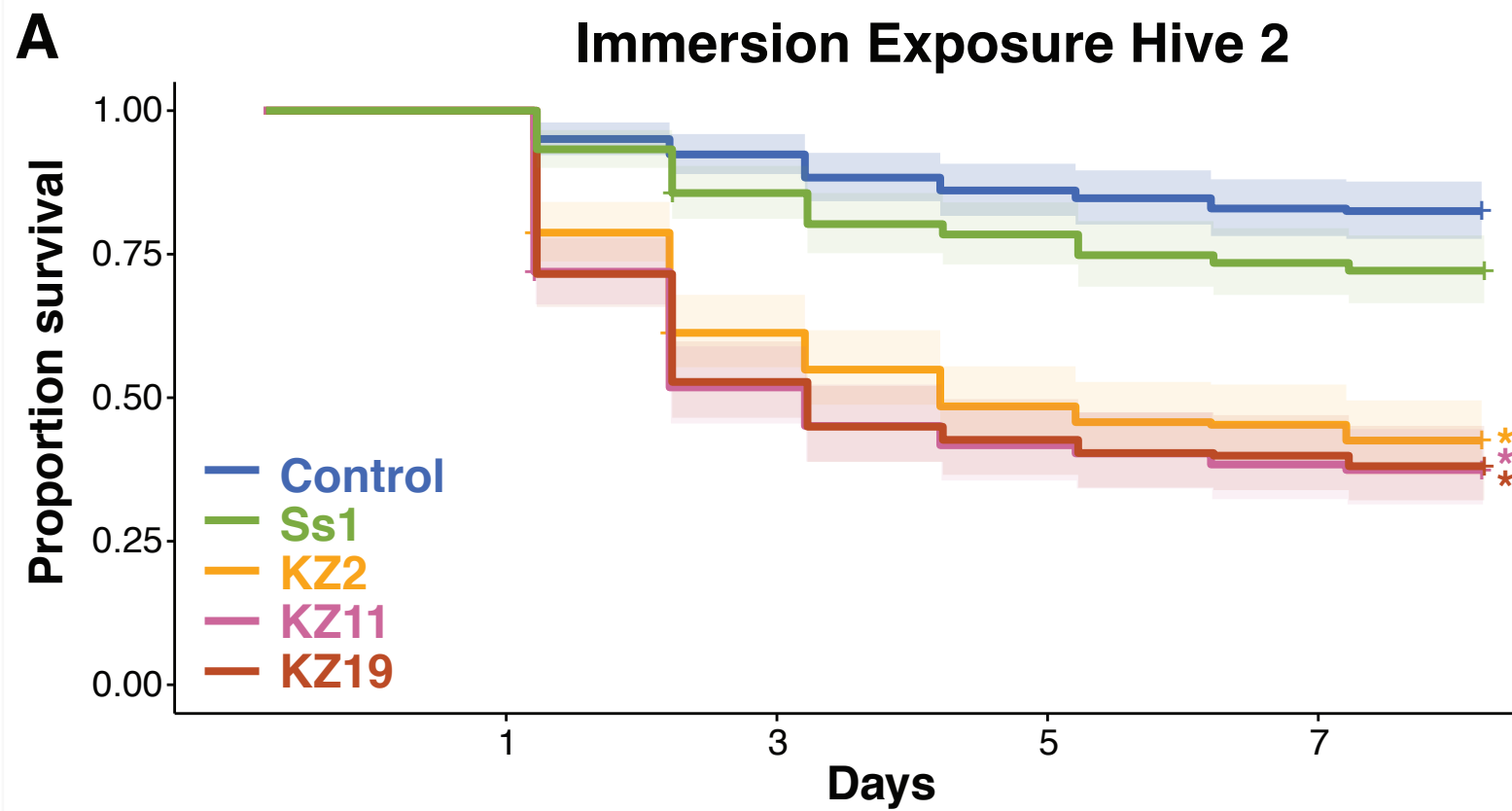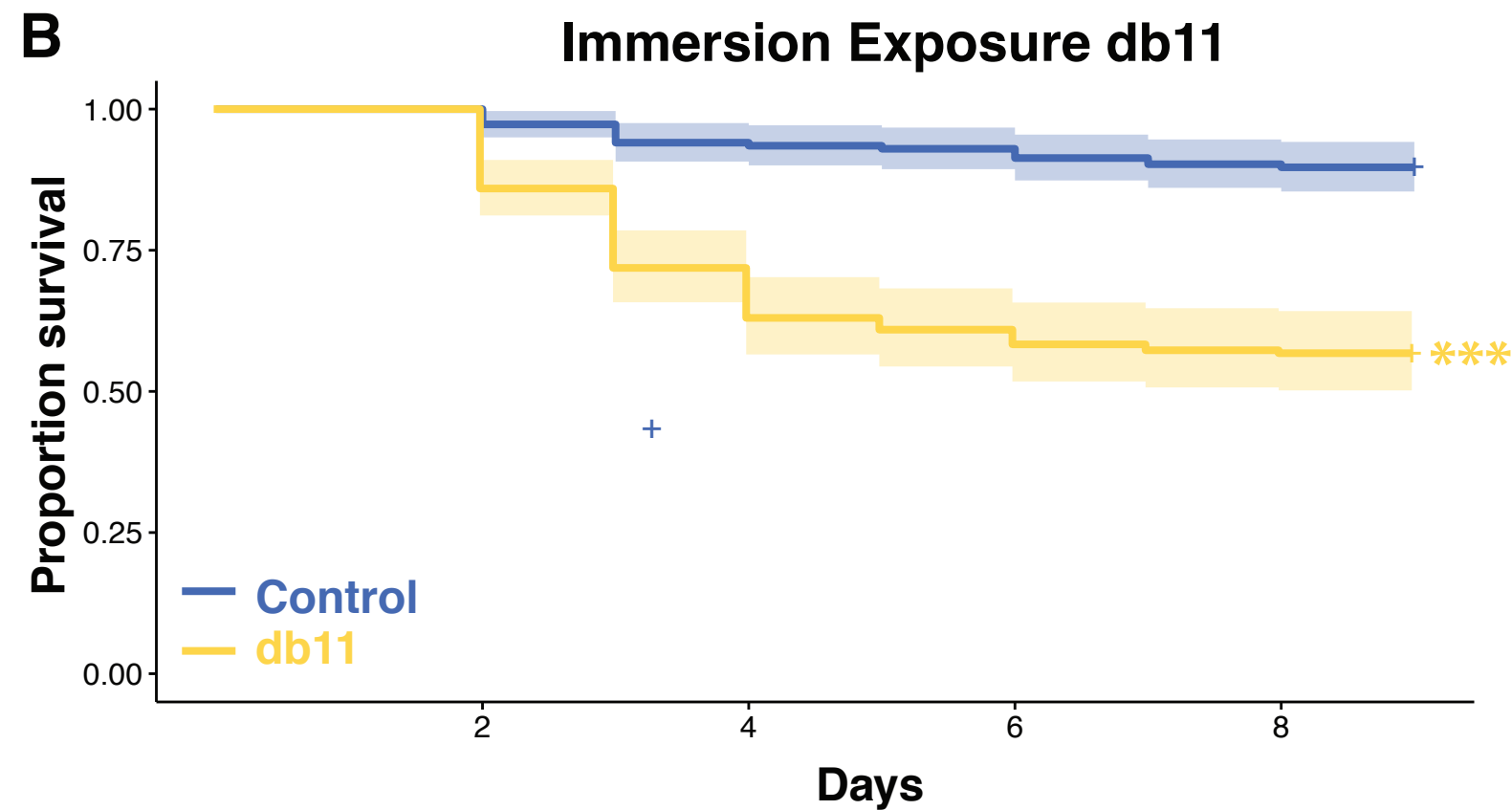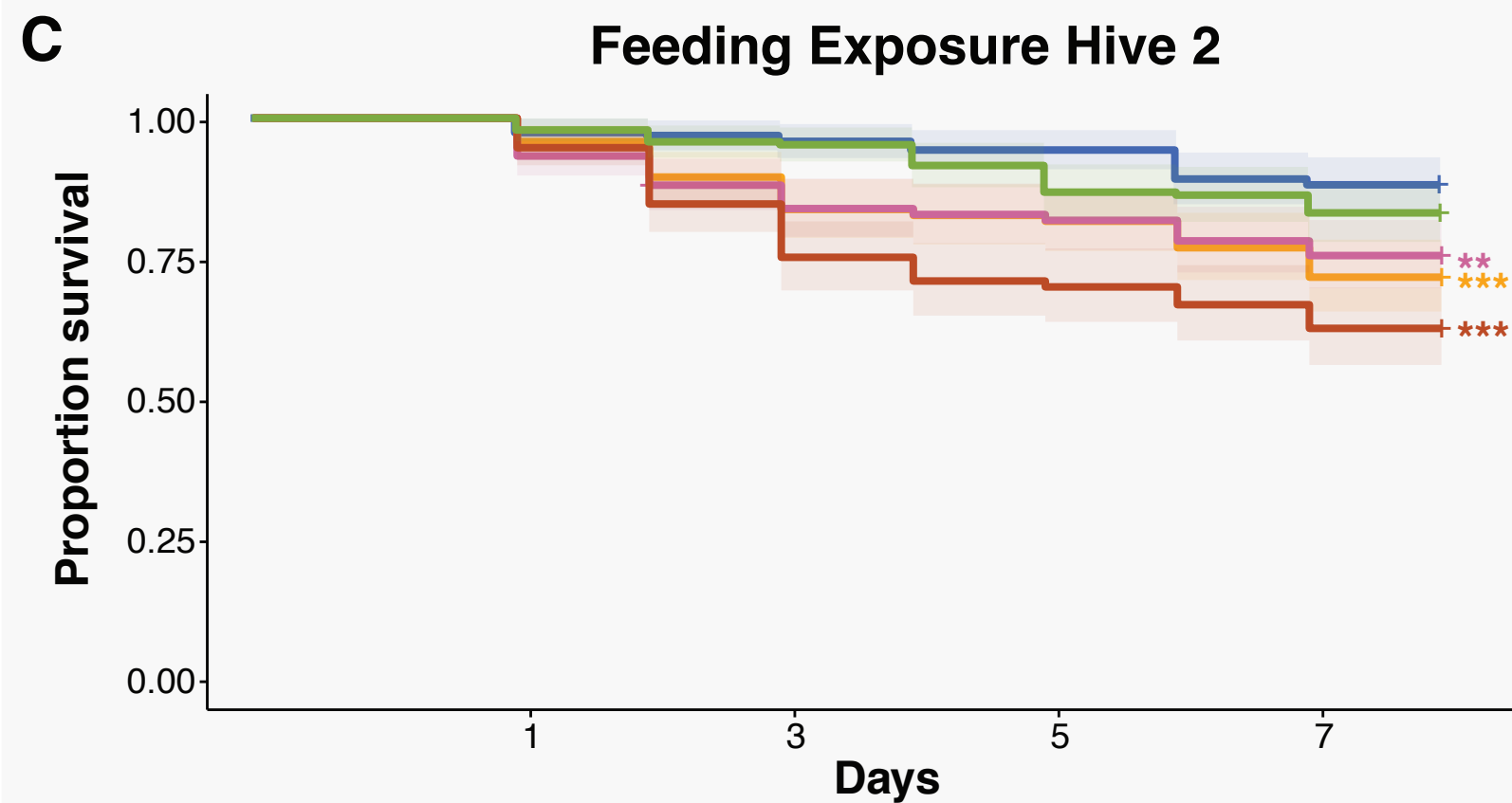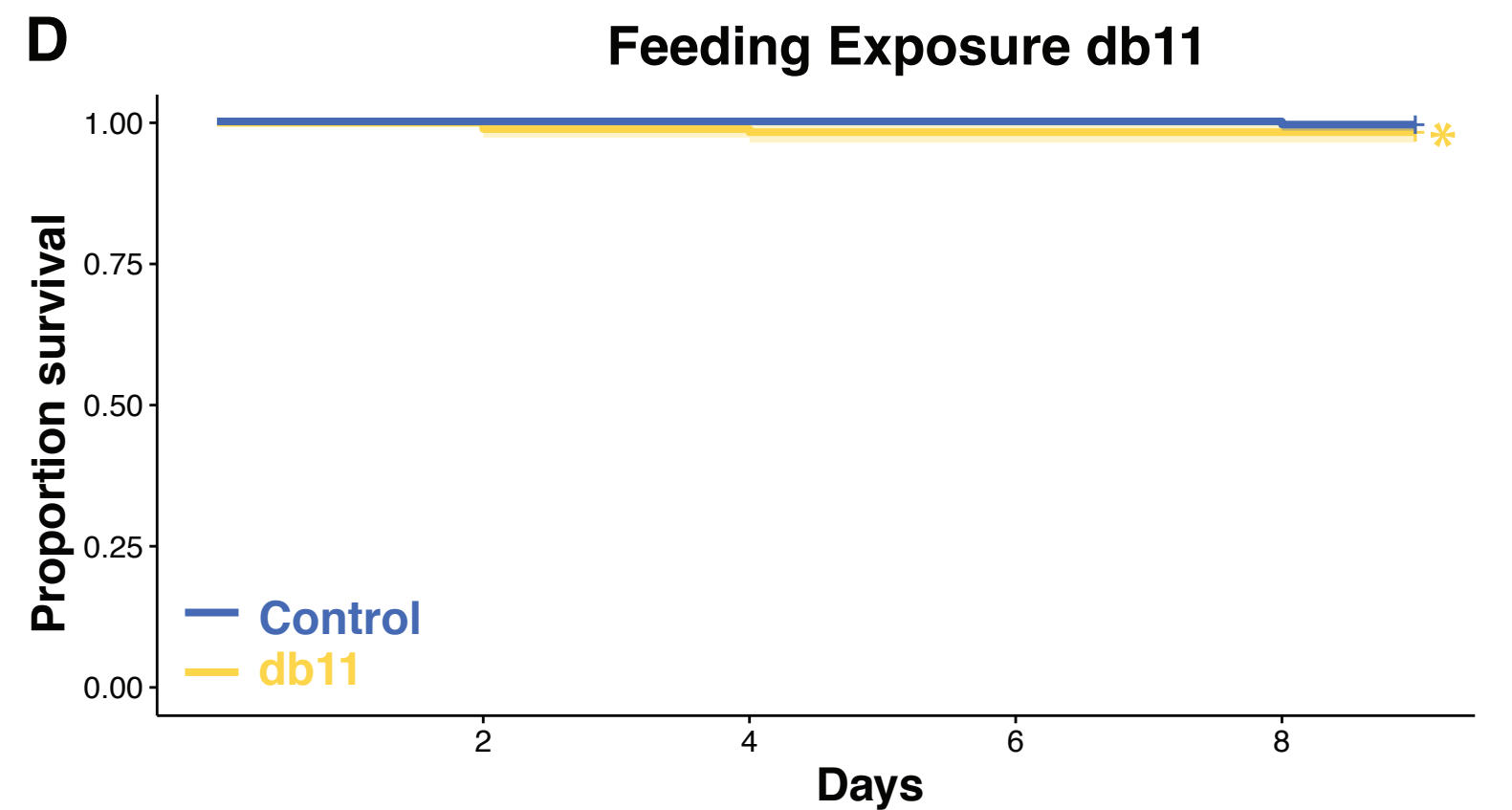

Supplement: FIG S2 [file mbo005184101sf2.pdf]

## Hemolymph Injections Hive 2

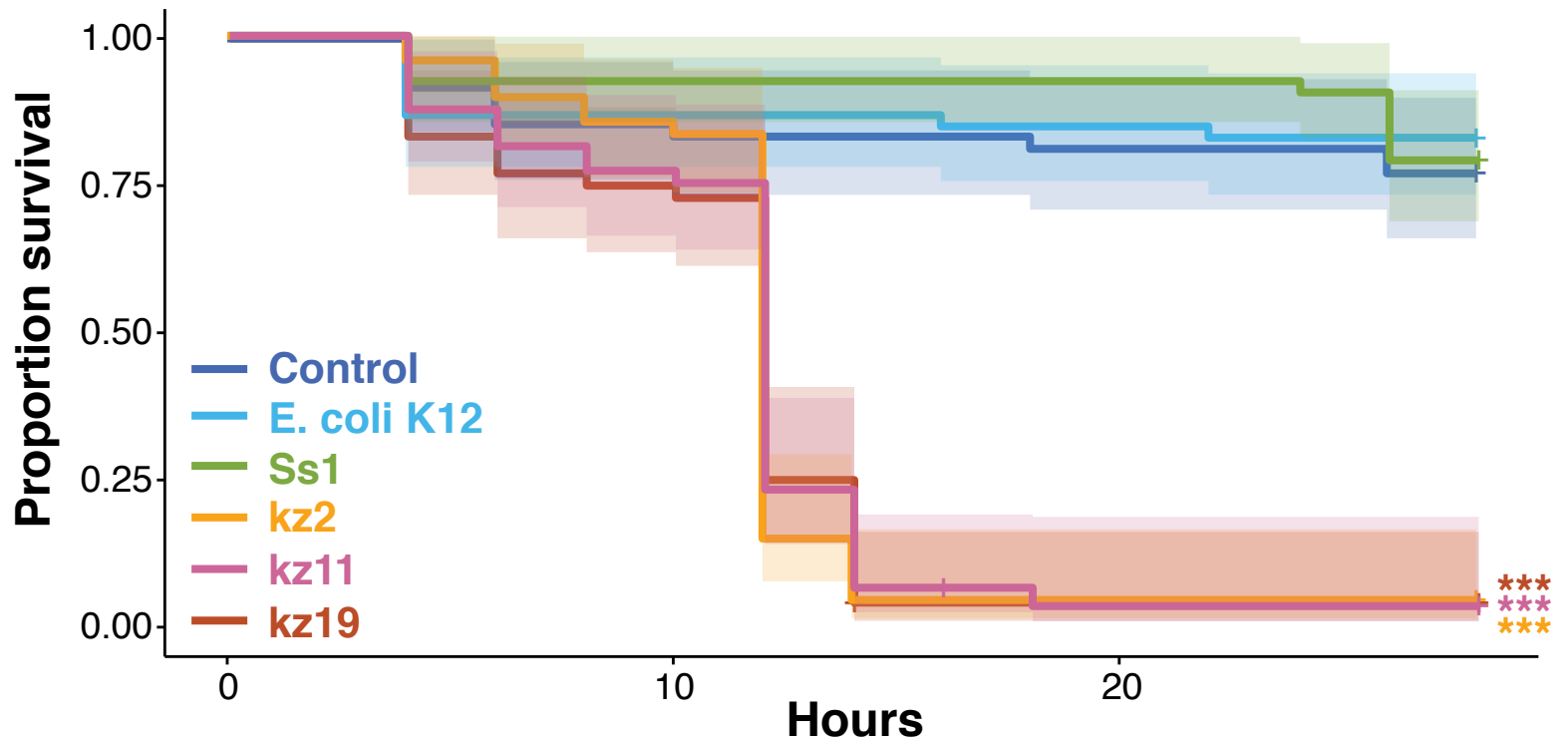

## Hemolymph Injections db11

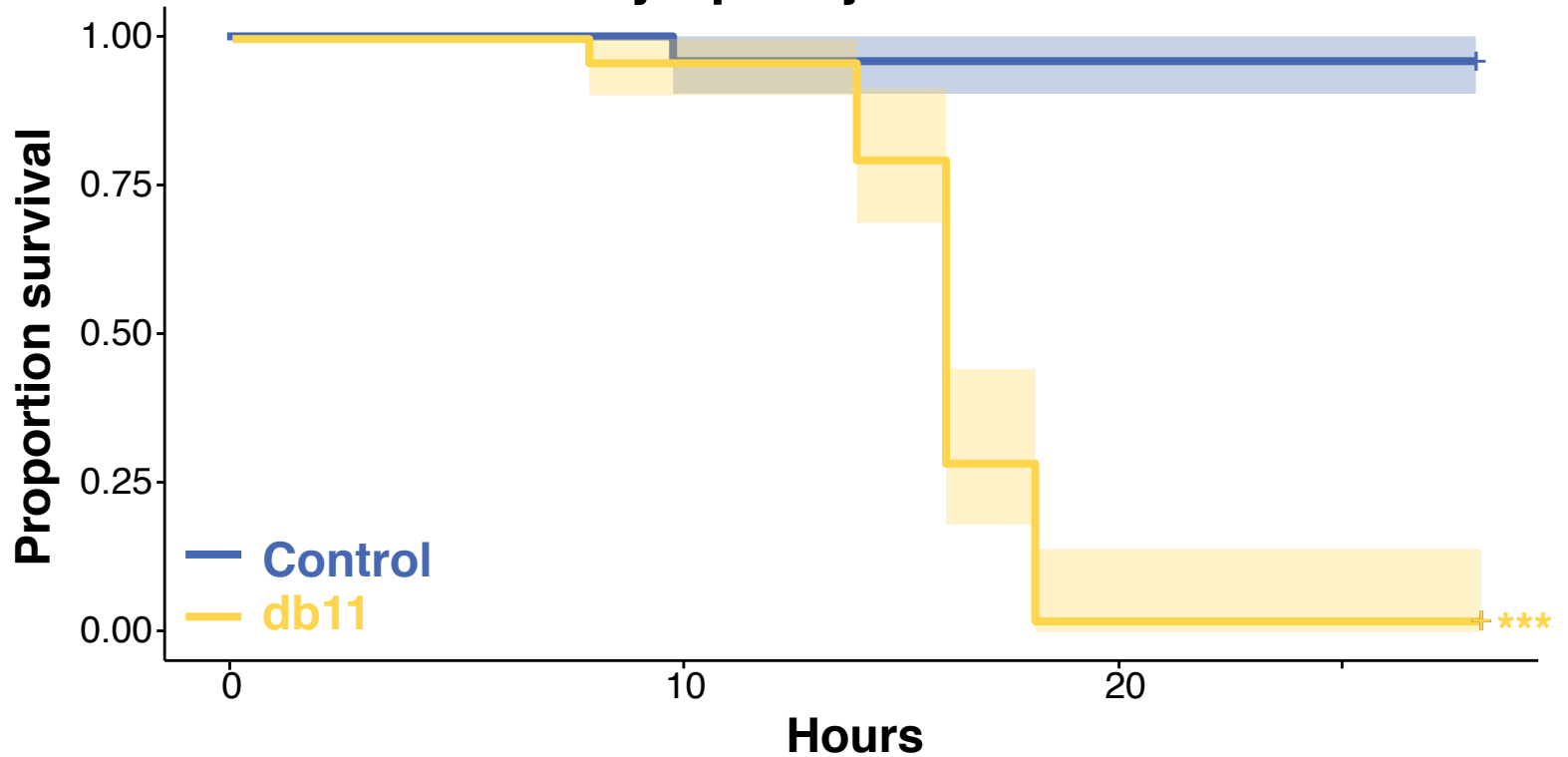

Supplement: FIG S3 [file mbo005184101sf3.pdf]

# Growth Rate

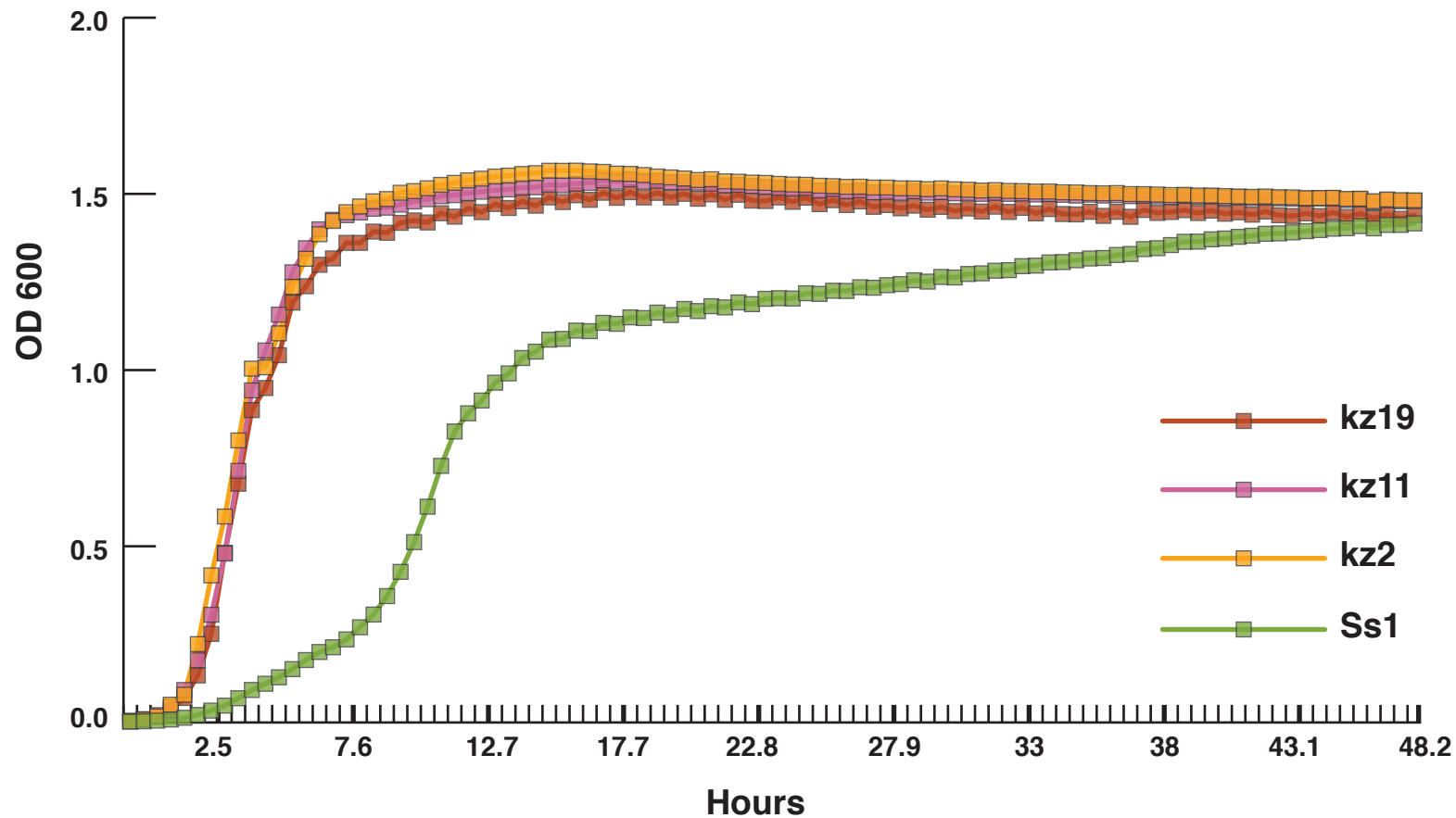

Supplement: FIG S4 [file mbo005184101sf4.pdf]

A

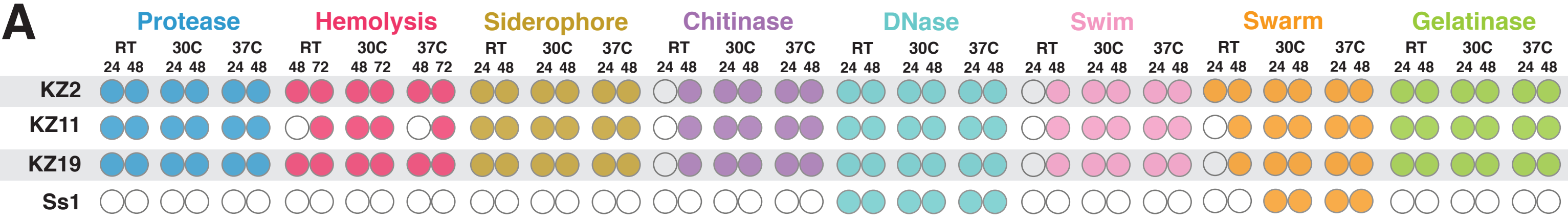

B

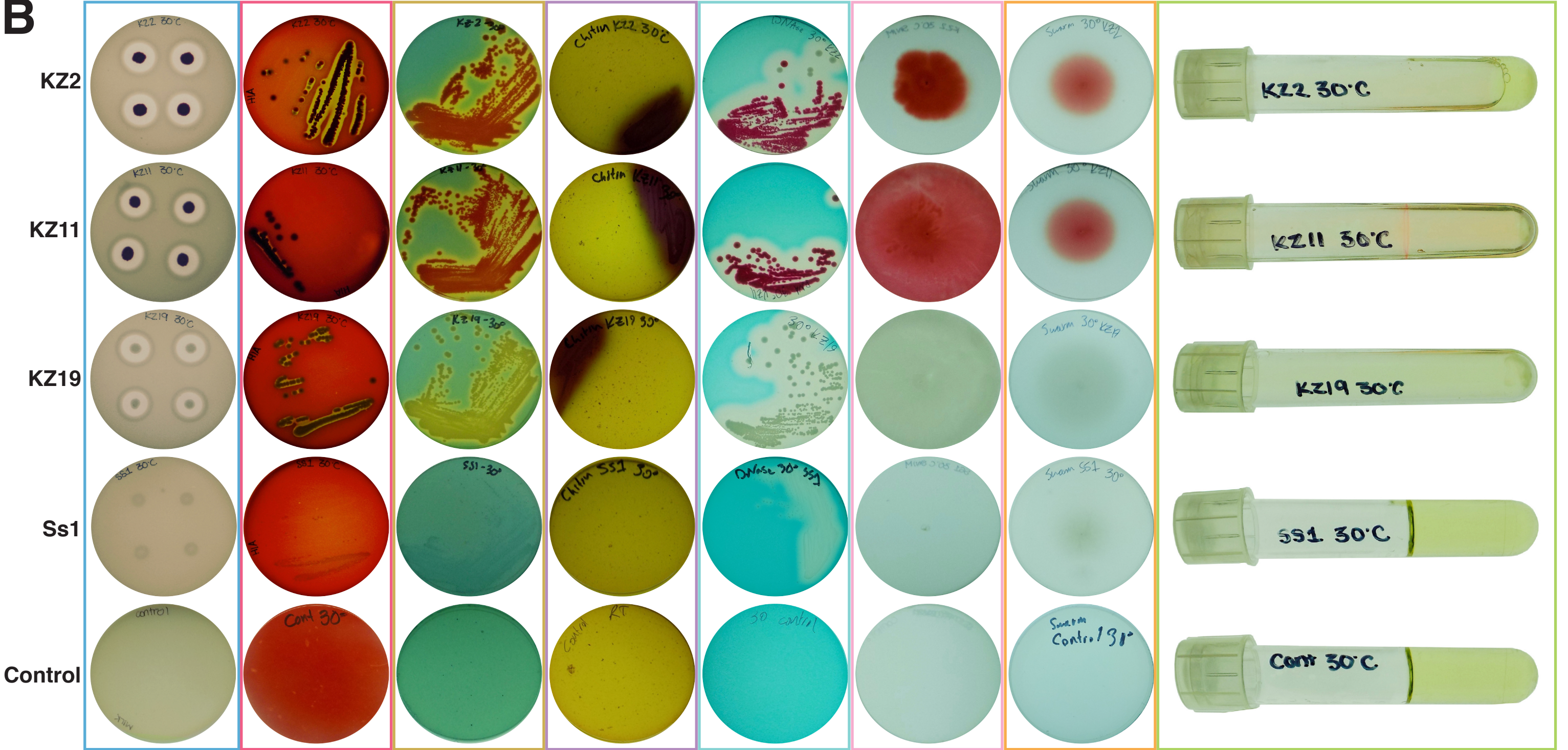

Supplement: FIG S5 [file mbo005184101sf5.pdf]

Region 4

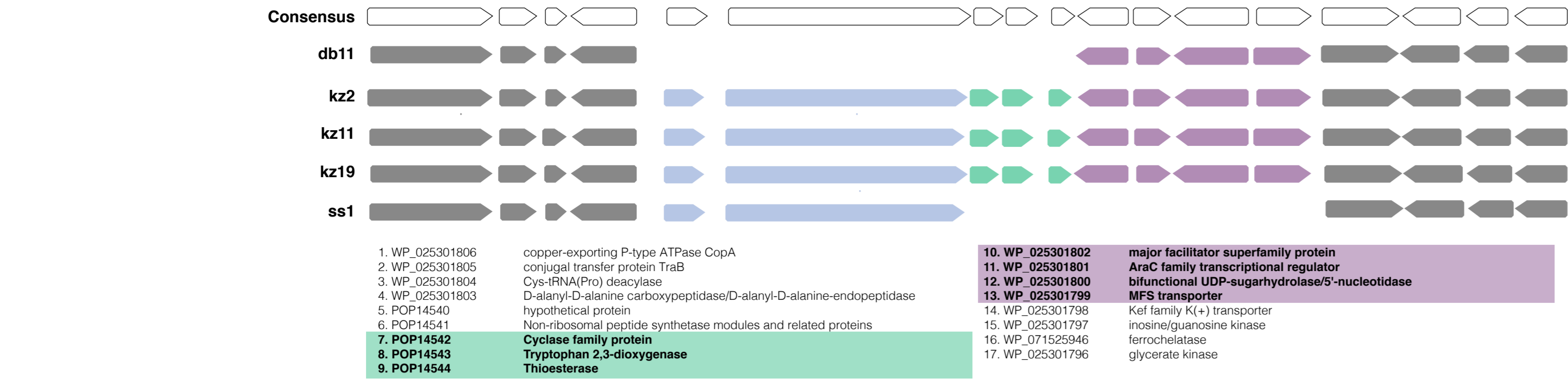

Region 5

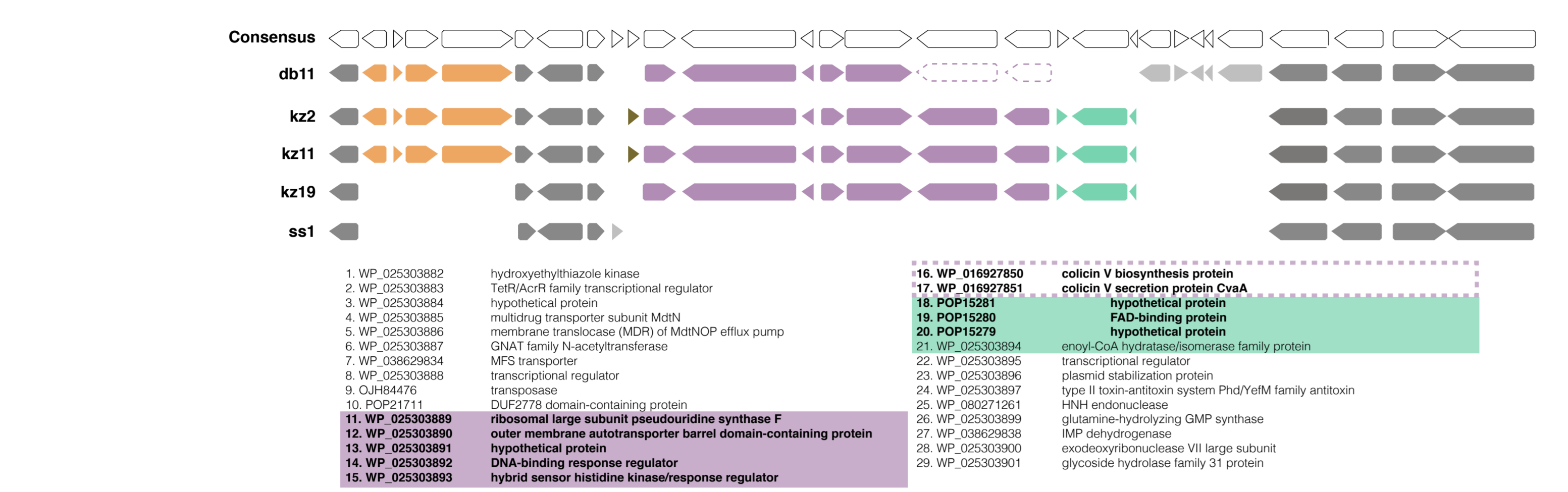

Region 6

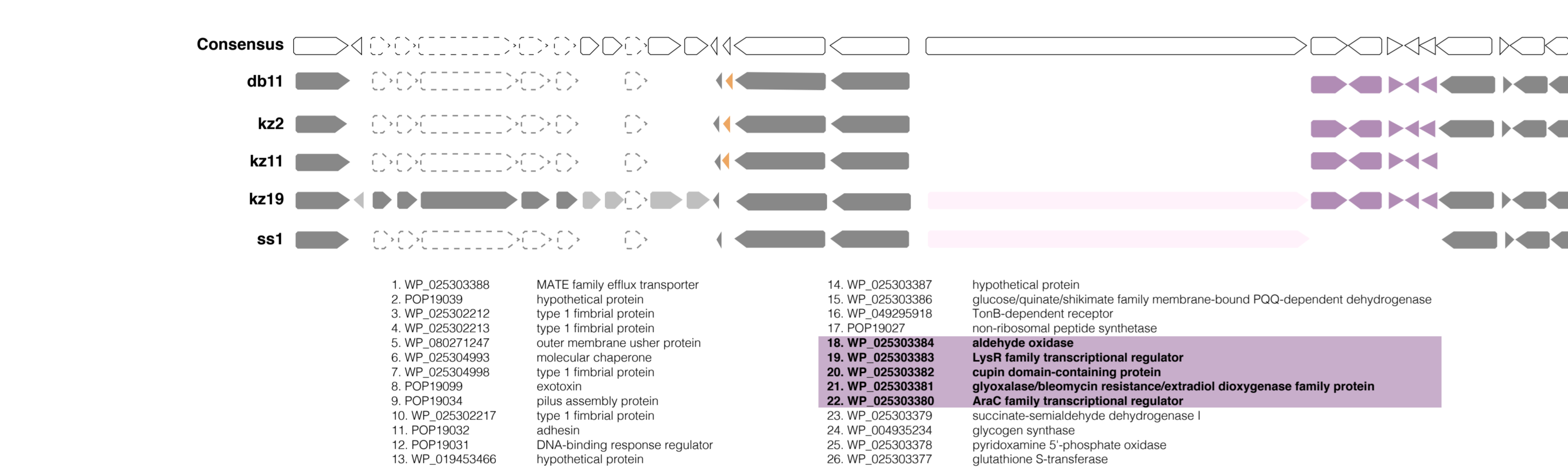

Region 7

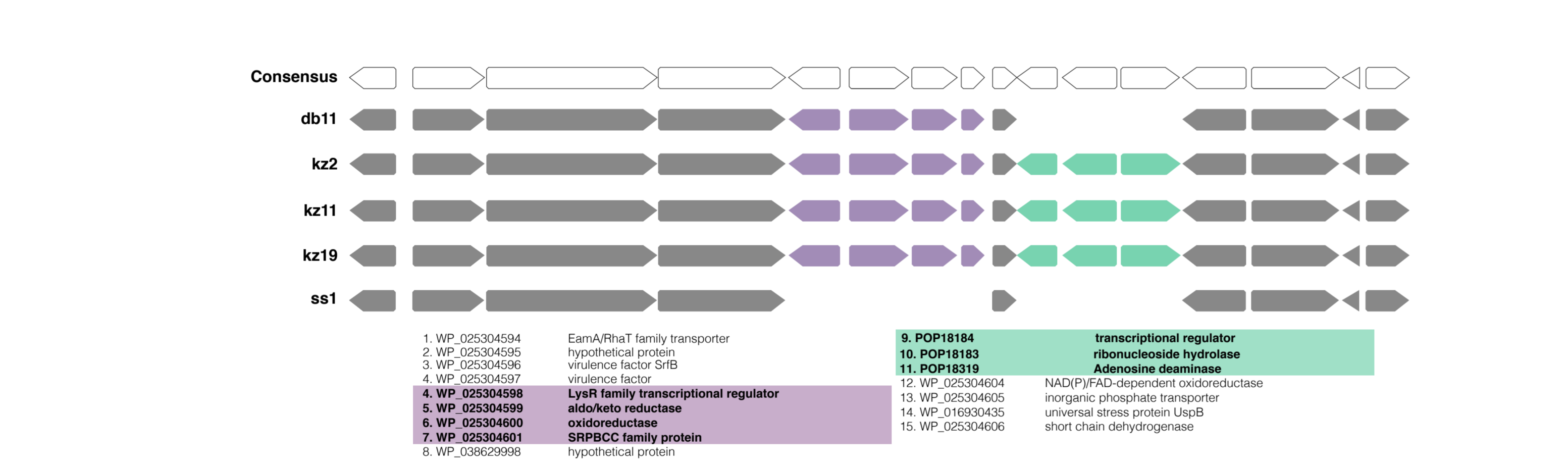

Region 8

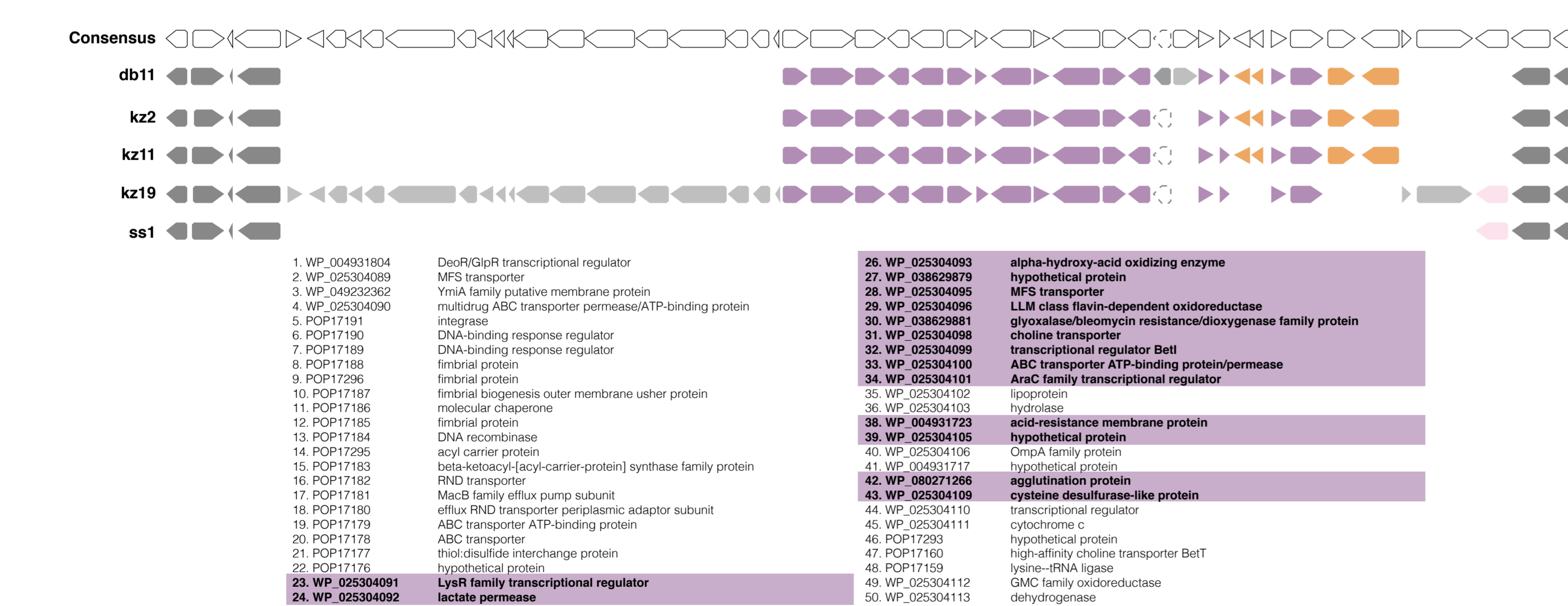

Region 9

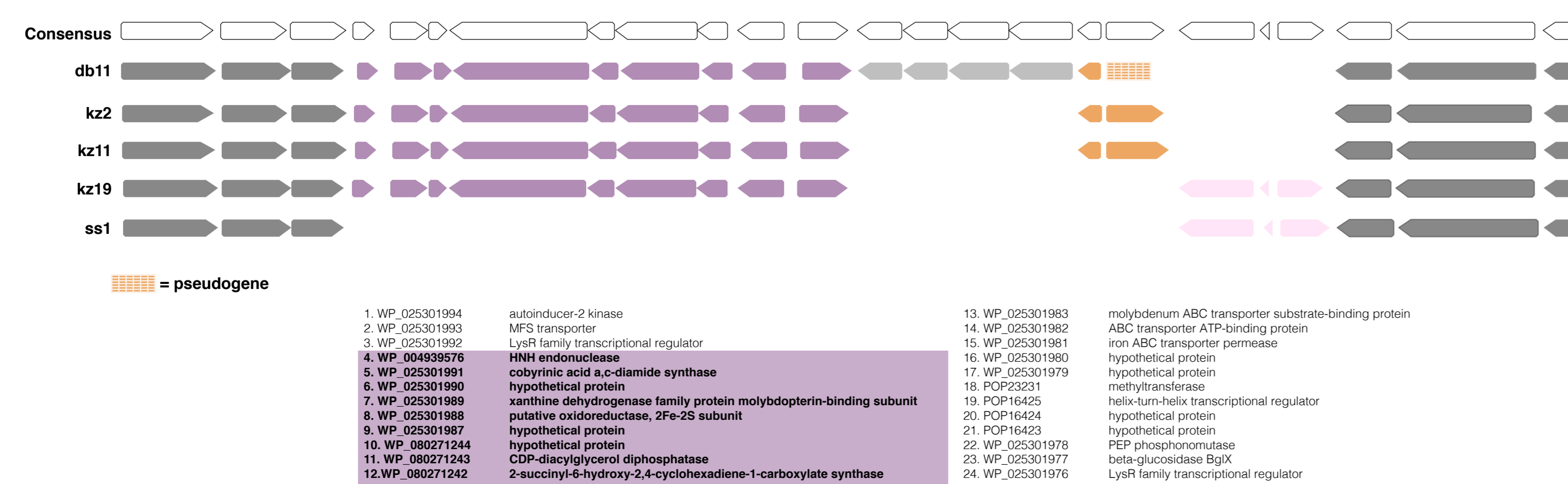

Region 10

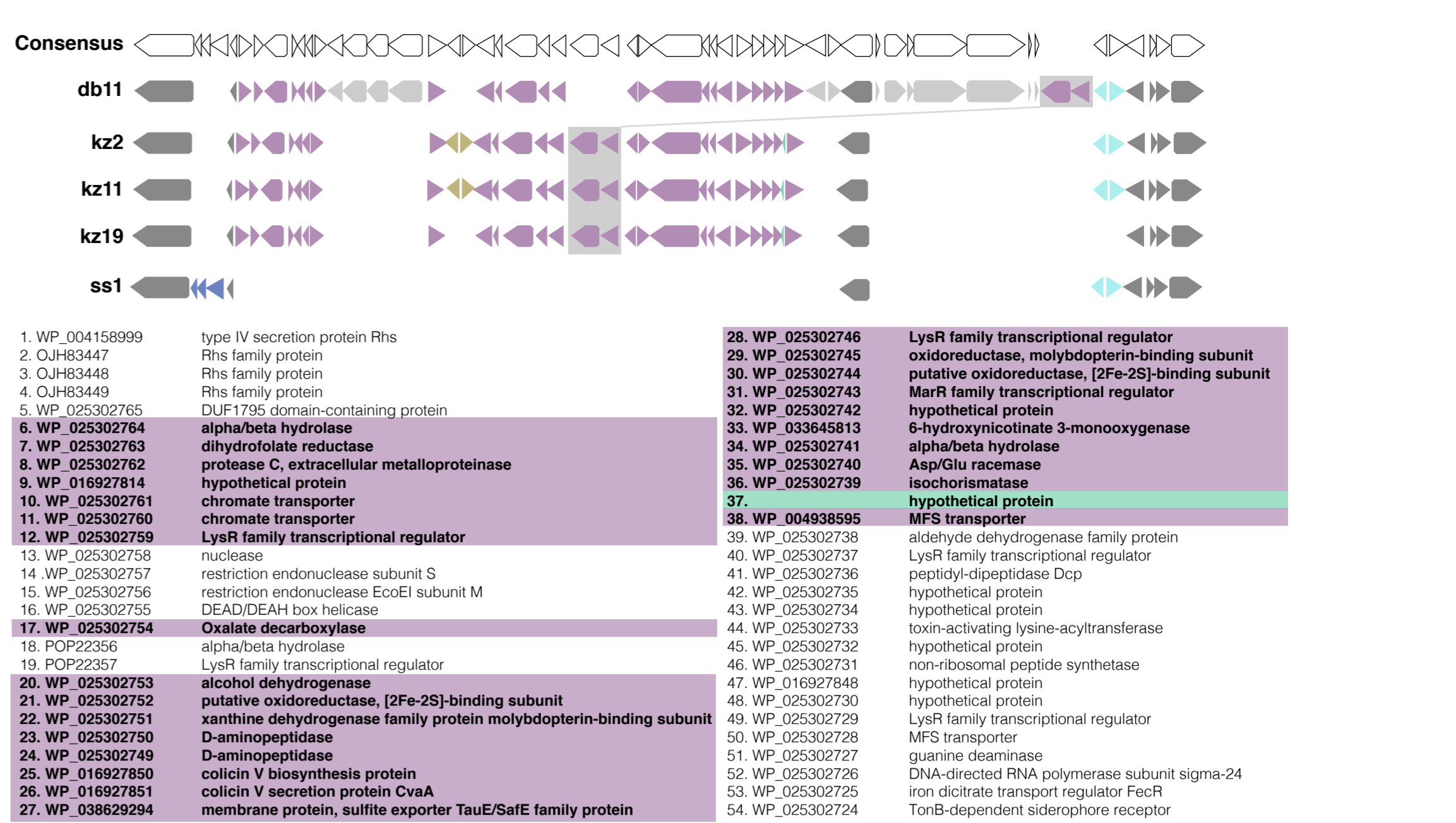

Region 11

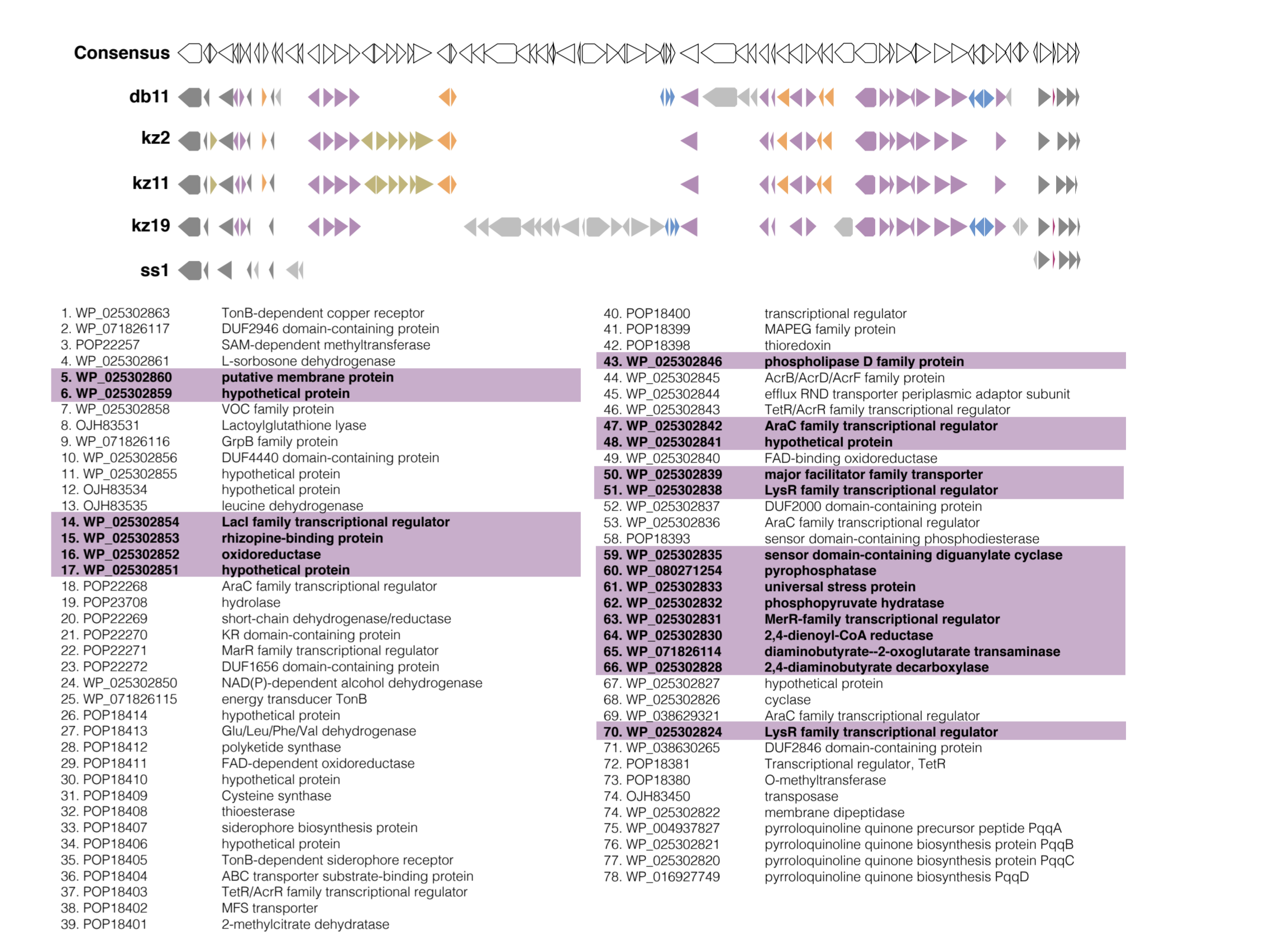

Region 12

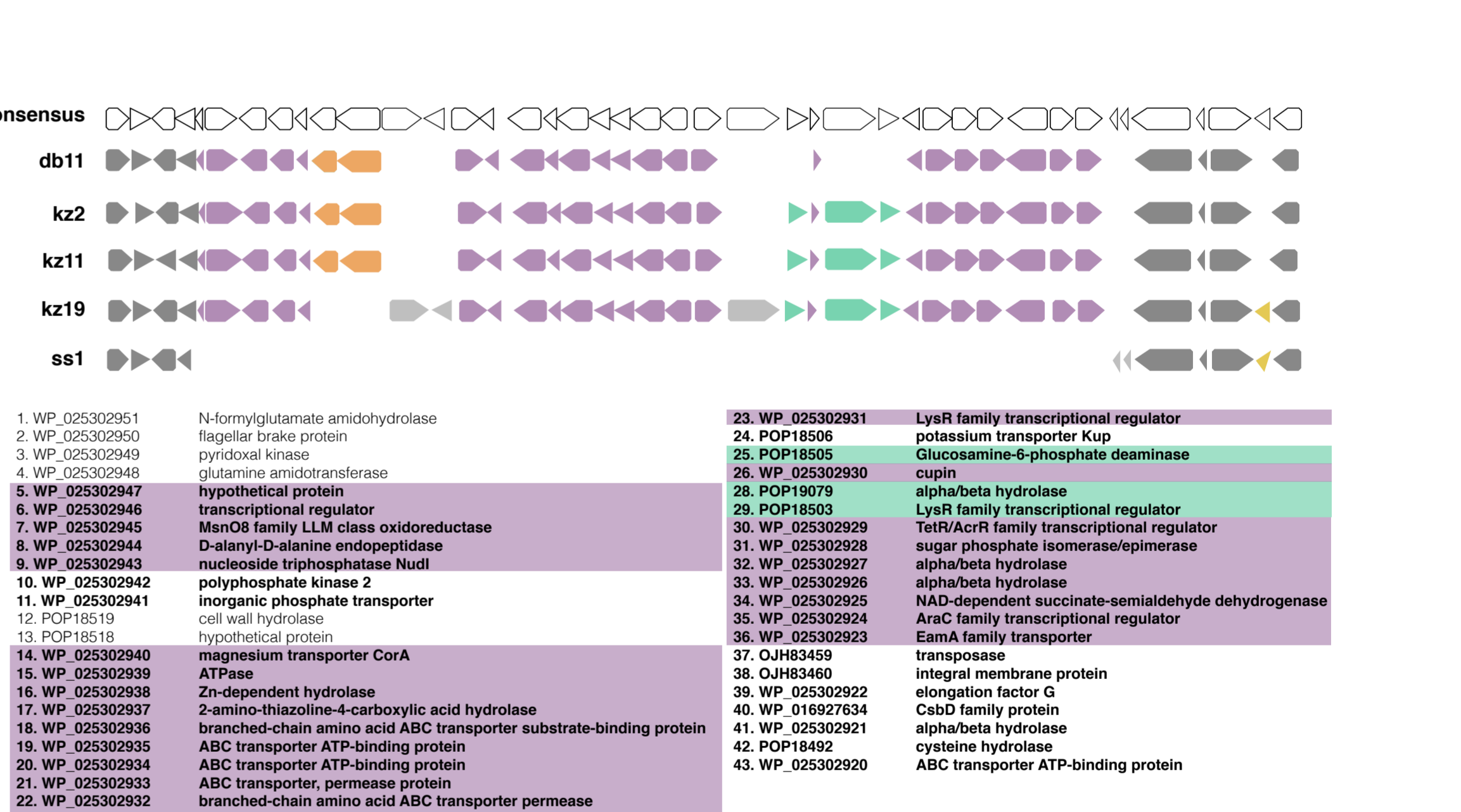

Region 13

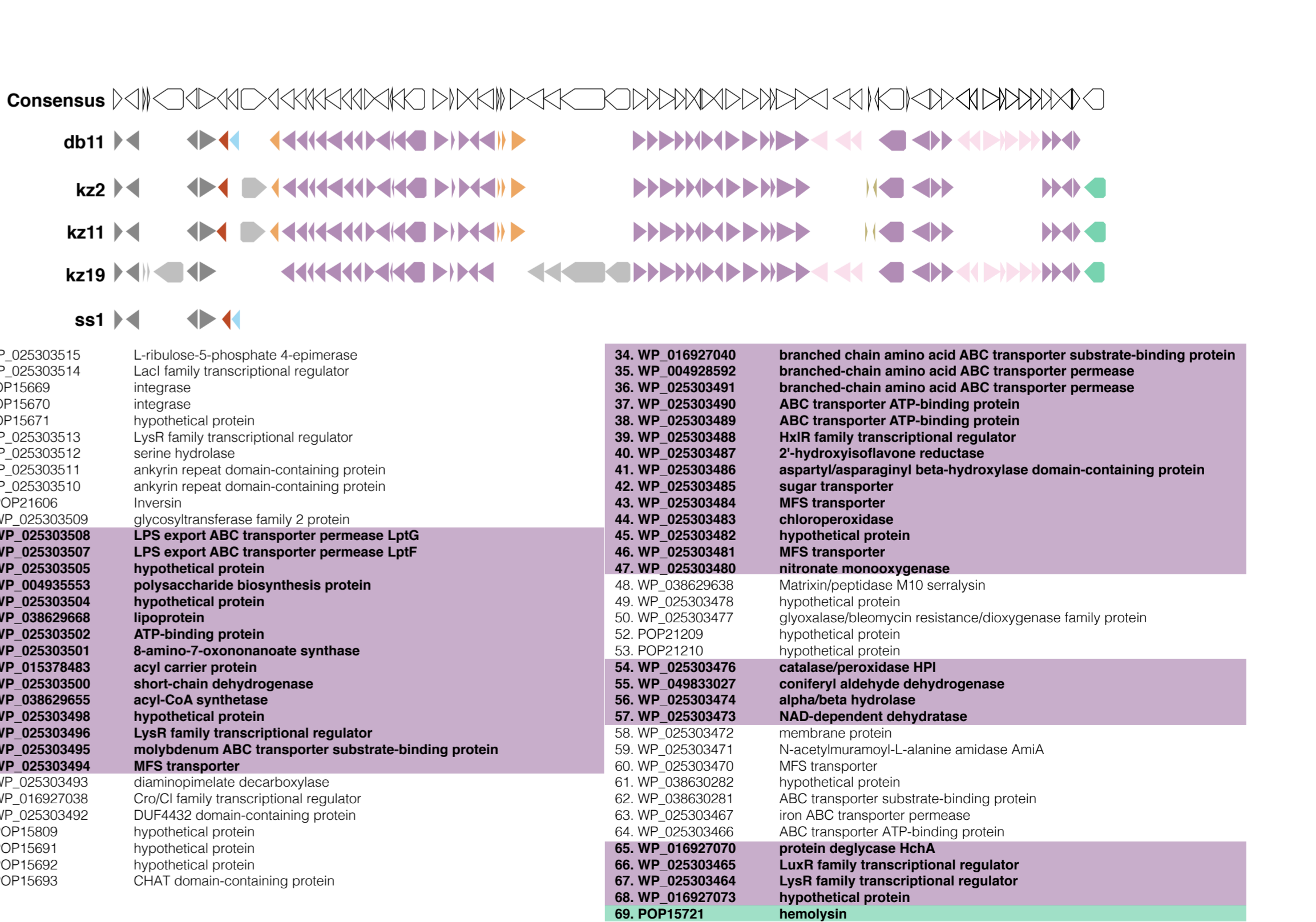

Supplement: FIG S7 [file mbo005184101sf7.pdf]
