# Supplementary material for: Pathogenicity of Serratia marcescens Strains in Honey Bees
Source: mBio. 2018 Oct 9;9(5):e01649-18. doi: 10.1128/mBio.01649-18 (PMC6178626; doi:10.1128/mBio.01649-18)
Supplement: TABLE S2 [file mbo005184101st2.pdf]

Table S2. Genome assembly and quality data for the S. marcescens strains sequenced in this study.

| | Contigs | Avg contig len | Min contig len | Max contig len | Min coverage | Max coverage | Genome size | CDS | % GC | ANI to db11 |
|---|---|---|---|---|---|---|---|---|---|---|
| **KZ2** | 20 | 240,066 | 1220 | 2,786,944 | 81 | 3,610 | 5,035,724 | 4689 | 59.8 | 95.08 |
| **KZ11** | 21 | 186,739 | 1221 | 1,990,358 | 134 | 4,000 | 5,036,791 | 4691 | 59.8 | 95.08 |
| **KZ19** | 24 | 209,266 | 1221 | 842,184 | 52 | 1,532 | 5,226,072 | 4844 | 59.5 | 95.02 |
