# Supplementary material for: Pathogenicity of Serratia marcescens Strains in Honey Bees
Source: mBio. 2018 Oct 9;9(5):e01649-18. doi: 10.1128/mBio.01649-18 (PMC6178626; doi:10.1128/mBio.01649-18)
Supplement: FIG S6 [file mbo005184101sf6.pdf]

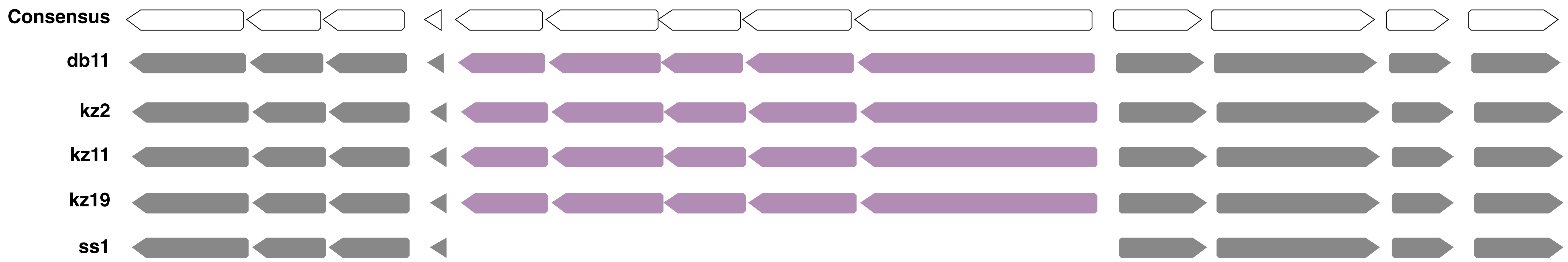

1. WP_025301910 molybdenum ABC transporter ATP-binding protein ModC
2. WP_019454005 molybdate ABC transporter permease subunit ModB
3. WP_025301909 molybdate ABC transporter substrate-binding protei
4. WP_004939813 multidrug efflux pump-associated protein, AcrZ family
5. **WP_025301908 ABC transporter ATP-binding protein**
6. **WP_025301907 iron ABC transporter permease**
7. **WP_025301906 class I SAM-dependent methyltransferase**
8. **WP_025301905 putative iron transport protein**
9. **WP_025301904 TonB-dependent receptor**
10. WP_025301903 molybdenum-dependent transcriptional regulator
11. WP_025301902 molybdate ABC transporter ATP-binding protein ModF
12. WP_025301901 cysteine hydrolase
13. WP_025301900 CPBP family intramembrane metalloprotease

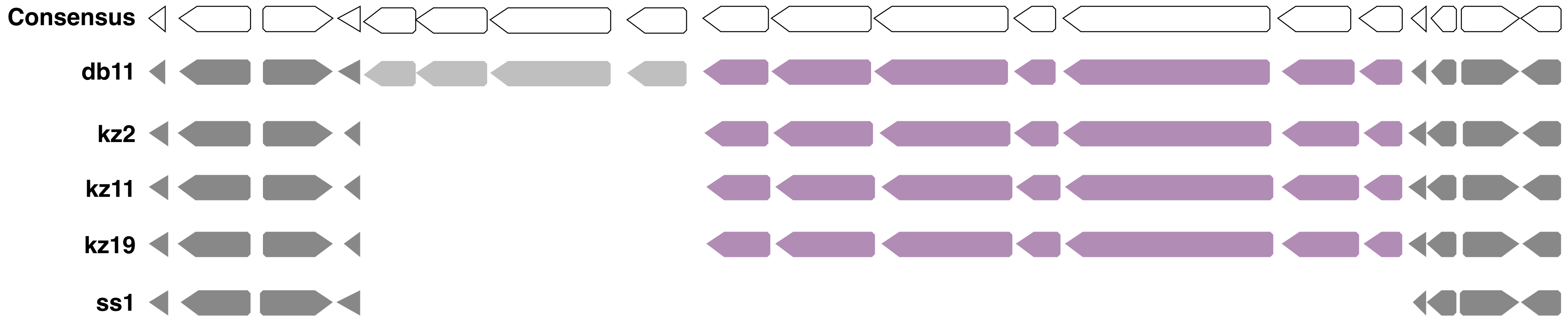

1. WP_025301735 type II toxin-antitoxin system prevent-host-death family antitoxin
2. WP_025301734 fructokinase
3. WP_004940461 recombination-associated protein RdgC
4. WP_004940463 pyrimidine/purine nucleoside phosphorylase
5. WP_025301733 hypothetical protein
6. WP_025301732 DUF1177 domain-containing protein
7. WP_025301731 membrane protein
8. WP_025301730 IclR family transcriptional regulator
9. **WP_025301729 energy transducer TonB**
10. **WP_025301728 HlyD family type I secretion periplasmic adaptor subunit**
11. **WP_025301727 type I secretion system permease/ATPase**
12. **WP_025301726 hemophore HasA**
13. **WP_025301725 TonB-dependent heme receptor**
14. **WP_025301724 iron dicitrate transport regulator FecR**
15. **WP_025301723 RNA polymerase sigma factor**
16. WP_004940491 hypothetical protein
17. WP_025301722 nuclear transport factor 2 family protein
18. WP_025301721 AraC family transcriptional regulator
19. WP_025301720 shikimate kinase AroL
